# Healthy Lifestyle Care vs Guideline-Based Care for Low Back Pain: A Randomized Clinical Trial

**DOI:** 10.1001/jamanetworkopen.2024.53807

**Published:** 2025-01-10

**Authors:** Emma Mudd, Simon R. E. Davidson, Steven J. Kamper, Priscilla Viana da Silva, Connor Gleadhill, Rebecca Kate Hodder, Robin Haskins, Bruce Donald, Christopher M. Williams

**Affiliations:** 1 University Centre for Rural Health, School of Health Sciences, University of Sydney, Lismore, New South Wales, Australia; 2 Population Health, Hunter New England Local Health District, Wallsend, New South Wales, Australia; 3 School of Health Sciences, University of Sydney, Camperdown, New South Wales, Australia; 4 Nepean Blue Mountains Local Health District, Penrith, New South Wales, Australia; 5 Hunter Medical Research Institute, New Lambton Heights, New South Wales, Australia; 6 School of Medicine and Public Health, University of Newcastle, Callaghan, New South Wales, Australia; 7 John Hunter Hospital Outpatient Services, New Lambton Heights, New South Wales, Australia; 8 John Hunter Hospital Physiotherapy Department, New Lambton Heights, New South Wales, Australia; 9 Research and Knowledge Translation Directorate, Mid North Coast Local Health District, Port Macquarie, New South Wales, Australia

## Abstract

**Importance:**

An unhealthy lifestyle is believed to increase the development and persistence of low back pain, but there is uncertainty about whether integrating support for lifestyle risks in low back pain management improves patients’ outcomes.

**Objective:**

To assess the effectiveness of the Healthy Lifestyle Program (HeLP) compared with guideline-based care for low back pain disability.

**Design, Setting, and Participants:**

This superiority, assessor-blinded randomized clinical trial was conducted in Australia from September 8, 2017, to December 30, 2020, among 346 participants who had activity-limiting chronic low back pain and at least 1 lifestyle risk (overweight, poor diet, physical inactivity, and/or smoking), referred from hospital, general practice, and community settings. Statistical analysis was performed from January to December 2021.

**Interventions:**

Participants were block randomized to the HeLP intervention (n = 174; 2 postrandomization exclusions) or guideline-based physiotherapy care (n = 172), stratified by body mass index, using a concealed function in REDCap. HeLP integrated healthy lifestyle support with guideline-based care using physiotherapy and dietetic consultations, educational resources, and telephone-based health coaching over 6 months.

**Main Outcomes and Measures:**

The primary outcome was low back pain disability (Roland Morris Disability Questionnaire [RMDQ] score; 0-24 scale, where higher scores indicate greater disability) at 26 weeks. Secondary outcomes were weight, pain intensity, quality of life, and smoking. Analyses were performed by intention to treat. We estimated the complier average causal effect (CACE) as sensitivity analyses.

**Results:**

The sample of 346 individuals (mean [SD] age, 50.2 [14.4] years; 190 female participants [55%]) had a baseline mean (SD) RMDQ score of 14.7 (5.4) in the intervention group and 14.0 (5.5) in the control group. At 26 weeks, the between-group difference in disability was −1.3 points (95% CI, −2.5 to −0.2 points; *P* = .03) favoring HeLP. CACE analysis revealed clinically meaningful benefits in disability among compliers, favoring HeLP (−5.4 points; 95% CI, −9.7 to −1.2 points; *P* = .01). HeLP participants lost more weight (−1.6 kg; 95% CI, −3.2 to −0.0 kg; *P* = .049) and had greater improvement in quality of life (physical functioning score; 1.8, 95% CI, 0.1-3.4; *P* = .04) than control participants.

**Conclusions and Relevance:**

Combining healthy lifestyle management with guideline-based care for chronic low back pain led to small improvements in disability, weight, and quality of life compared with guideline-based care alone, without additional harm. Targeting lifestyle risks in the management of chronic low back pain may be considered safe and may offer small additional health benefits beyond current guideline-based care.

**Trial Registration:**

http://anzctr.org.au Identifier: ACTRN12617001288314

## Key Points

**Question:**

What is the effect of integrating healthy lifestyle care into back pain management on low back pain disability compared with current guideline-recommended care?

**Findings:**

In this randomized clinical trial with 346 participants, there was a greater mean reduction in disability favoring the healthy lifestyle approach compared with guideline care alone, equivalent to a mean difference of −1.3 points out of 24. Participants in the healthy lifestyle group who complied with treatment had large clinical meaningful improvements in disability.

**Meaning:**

This study suggests that lifestyle care can safely be integrated into care for chronic low back pain, providing small improvements in disability compared with current guideline-recommended care and an opportunity to concurrently address prevalent chronic disease risks in this patient group.

## Teaser

This randomized clinical trial assesses the effectiveness of the Healthy Lifestyle Program compared with guideline-based care for low back pain disability.

## Introduction

Low back pain is a leading cause of disability globally and a significant public health problem.^1,2^ Observational studies have linked the development and persistence of low back pain with lifestyle risk factors, such as being overweight, smoking, physical inactivity, and poor diet.^3,4,5,6^ Disability associated with back pain can also lead to compensatory unhealthy lifestyle behaviors, exposing people to increased risk of chronic diseases.^7^

Despite associations between lifestyle risks and back pain, the effectiveness of targeting lifestyle to manage back pain is uncertain.^8,9,10^ Based on a recent systematic review of weight management interventions, the World Health Organization global guideline for low back pain does not recommend weight loss due to very-low-certainty evidence from small studies with high risk of bias.^8,9,10,11^ No trials of smoking interventions for back pain have been reported,^12^ and while exercise is considered a core treatment for low back pain,^13,14,15^ trials targeting sedentary lifestyle are lacking.^16^

Internationally, integrated health care for people experiencing multiple health challenges is endorsed by governments and health policy, acknowledging the associations between chronic diseases and their determinants.^17^ Integrated care aims to support a person-centered approach to connect prevention, treatment, and rehabilitation actions, according to an individual’s needs.^17^ Currently, there is limited evidence for the benefits of integrating lifestyle-focused support into low back pain care, and few people with these coexisting health challenges receive integrated care.^11,18^

In response to knowledge gaps about integrated care for low back pain and unhealthy lifestyle, we designed the HeLP (Healthy Lifestyle Program) for Chronic Low Back Pain Trial.^19^ The HeLP trial aimed to assess the benefits and potential harms of integrating management of unhealthy lifestyle with guideline-based care on low back pain disability compared with current guideline-recommended care alone.

## Methods

### Trial Design and Oversight

The HeLP trial was a pragmatic (in terms of eligibility, setting, recruitment, and intervention delivery and organization^20^), 1:1 randomized clinical multisite superiority trial conducted from September 8, 2017, to December 30, 2020, to compare the HeLP intervention (guideline and healthy lifestyle care) with guideline-based care. Trial findings were reported using the Consolidated Standards of Reporting Trials (CONSORT) reporting guideline and statement and International Committee of Medical Journal Editors recommendations. The trial was approved by the Hunter New England Research Ethics Committee and the University of Newcastle Human Research Ethics Committee and was overseen by a trial steering committee of trial investigators, a research team, and health service representatives. Participants provided written informed consent. The trial protocol was published in 2019,^19^ and a prespecified statistical analysis plan was submitted for publication September 2020,^21^ before data collection ended in December 2020. The trial protocol and statistical analysis plan are in Supplement 1.

### Participants

We recruited adults with nonspecific chronic low back pain (≥3 months’ duration), moderate pain intensity or activity limitation, and at least 1 lifestyle risk factor (overweight, not meeting recommended amount of physical activity or fruit and/or vegetable consumption, or smoking). Detailed eligibility criteria can be found in the eAppendix in Supplement 2 and trial protocols (Supplement 1).^19,21^

We took referrals from hospital outpatient services (ie, people discharged from neurosurgery and orthopedic services after 1 consultation with a specialist), from general practice, or directly from those responding to social media advertising. Eligibility, initial consent, and baseline data were assessed using computer-assisted telephone interviews with data entered directly into a REDCap database.^22^

### Randomization and Masking

Participants were randomized in a 1:1 ratio to intervention or control in permuted 6:4 blocks, to ensure equal distribution of body mass index (BMI; calculated as weight in kilograms divided by height in meters squared) categories (18.5-24.9, ≥25-29.9, and ≥30). Randomization was concealed in REDCap based on a randomization schedule generated by an independent statistician.^19^

To minimize performance bias, participants were not informed of treatment specifics until the initial consultation, only that they would receive 1 of 2 types of physiotherapy. Clinicians were assigned to deliver either the HeLP intervention or the control intervention, not both, at different sites. Follow-up data were collected using online or mailed surveys or by trained telephone interviewers blinded to treatment allocation. Analyses were conducted by independent statisticians using dummy coded variables for treatment group, per the published statistical analysis plan.^21^

### Interventions

The HeLP and guideline-based care interventions are described extensively elsewhere^19^ using the TIDieR (Template for Intervention Description and Replication) checklist.^23^ Treatments were delivered in hospital outpatient physiotherapy departments or a research institute clinic.

#### HeLP Intervention

**Figure 1.**
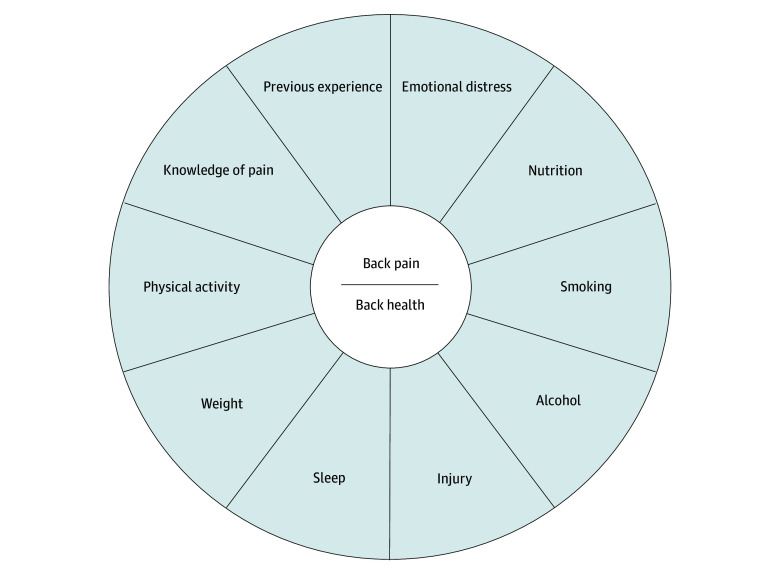
Healthy Lifestyle Program (HeLP) Model of Care Communication and Decision Aid

The HeLP intervention included guideline-based care plus back pain–specific healthy lifestyle education and support provided in clinical consultations, educational resources (booklet and web portal access), and telephone-based health coaching. Clinical consultations included up to 4 physiotherapist sessions and 1 dietitian session over 12 weeks, focusing on pain education lifestyle changes and self-management plans. Physiotherapy consultations aimed to generate conceptual change regarding pain beliefs, introduce lifestyle as an influence on pain, and develop an individualized self-management plan for appropriate lifestyle targets. Lifestyle targets were systematically introduced and discussed using a visual communication and decision aid (Figure 1). Dietitians predominantly (but not exclusively) focused on advice to improve diet quality, including alcohol consumption and specific weight management advice. Consultations used motivational interviewing and cognitive behavioral therapy and incorporated behavior change techniques.

Educational resources included a booklet and web portal with interactive information content. Telephone health coaching was provided by the New South Wales (NSW) Get Healthy Service, offering up to 10 tailored calls over 6 months for weight, physical activity, and diet coaching.^24^ Smokers were offered the NSW Quitline.^25^

HeLP intervention clinicians were trained in a 2-hour training workshop and a 2-day health behavior change course, and were clinically observed and received feedback on care delivered to initial patients. Get Healthy Service coaches received a 2-hour interactive training session to support alignment of coaching practice with clinical pain management. The session involved academic detailing for guideline-recommended low back pain care, evidence for associations between pain and lifestyle risks, and workshopping strategies for participants with pain to overcome barriers to coaching advice.

#### Control Intervention

Control group participants received guideline-informed care,^13^ including back pain education, advice, and exercise. Participants attended 3 consultations over 12 weeks. Control group clinicians did not provide lifestyle advice beyond exercise recommendations.

### Outcomes

Outcome measurement details and timing are provided eTable 1 in Supplement 2 and the published protocol.^19,21^ Baseline data included demographic data and pain characteristics. Follow-up data were completed at weeks 6, 12, 26, and 52 after randomization.

#### Primary Outcome

The primary outcome was disability at 26 weeks measured by the Roland Morris Disability Questionnaire (RMDQ) score, a widely recommended self-reported questionnaire (0-24 scale, with higher scores indicating greater disability) with strong clinometric properties.^26^

#### Secondary Outcomes

We prespecified 4 key secondary outcomes: mean back pain intensity (0-10 numerical rating scale, with 0 indicating no pain and 10 indicating the greatest possible pain),^27^ body weight (self-reported and objectively measured weight in kilograms),^28^ quality of life (12-item Short Form Health Survey V2),^29^ and smoking status (NSW Population Health Survey: current smoking status and number of cigarettes smoked per day).^30^

Exploratory outcomes, economic outcomes, and potential mediators included BMI, physical activity, diet quality, sleep quality, pain self-efficacy, alcohol consumption, psychological distress, self-reported health care, medication and services use, and work absenteeism and presenteeism. Process outcomes included intervention delivery (number of consultations and telephone calls, collected in routine logs by service providers), patient satisfaction, and reasons for withdrawals.

### Adverse Events

Adverse events were captured through open-text questions at consultations and follow-up. A trial steering committee monitored compliance, safety, and trial progress. A data monitoring committee, chaired by the principal investigator (C.M.W.), oversaw data integrity.

### Statistical Analysis

#### Sample Size

Statistical analysis was performed from January to December 2021. Using the Twisk method for mixed models,^31^ we calculated a sample size of 346 participants (173 per group), assuming a mean (SD) 3-point (5-point) difference on the RMDQ score,^32^ α of 5%, and 90% power. Statistical tests were 2-sided. The calculations accounted for repeated observations at 6, 12, and 26 weeks with an intracluster correlation of 0.5 and 18% loss to follow-up, but they ignored increases in statistical power due to stratification. The sample size also provided 80% power to detect a 2-point difference between prespecified subgroups of healthy weight vs overweight or obese for the RMDQ (moderation analysis).

#### Data Analysis

Analyses followed the prespecified statistical analysis plan.^21^ Data were analyzed using the intention-to-treat principle. The primary analysis used a mixed model for repeated measures (≤26 weeks) using data from all randomized participants with at least 1 follow-up and included fixed effects for time, group, baseline outcome values, stratification variables, and a time-by-group interaction. Effect modification (moderation analysis) of the primary outcome was assessed using a 3-way interaction term for time by group by baseline BMI (dichotomized; healthy BMI, ≥18.5-24.9; overweight, ≥25) and all relevant lower-order terms from the primary model.

Secondary and exploratory outcomes were analyzed using mixed models for repeated measures for continuous outcomes and logistic mixed-effects regression models for dichotomous outcomes. Fixed effects were as specified for the primary model. Secondary analyses of all outcomes at 52 weeks were conducted to assess long-term effects. The Fisher exact test was used to compare the incidence of adverse events between groups. We completed analyses using SAS, version 9.4 (SAS Institute Inc).^33^

#### Sensitivity Analyses

Three prespecified sensitivity analyses were completed.^21^ First, complier average causal effects (CACEs) were estimated using an instrumental variable regression approach.^34^ Compliance in the intervention group was defined as attending at least 2 consultations and completion of at least 5 health coaching calls (or agreed graduation). Second, the primary analysis was repeated, adjusting for prognostic variables (baseline variables considered prognostic with at least a 20% difference between groups). Third, multiple imputation assessed the effect of missing data using fully conditional specification predictive mean matching across 20 imputations.

## Results

**Figure 2.**
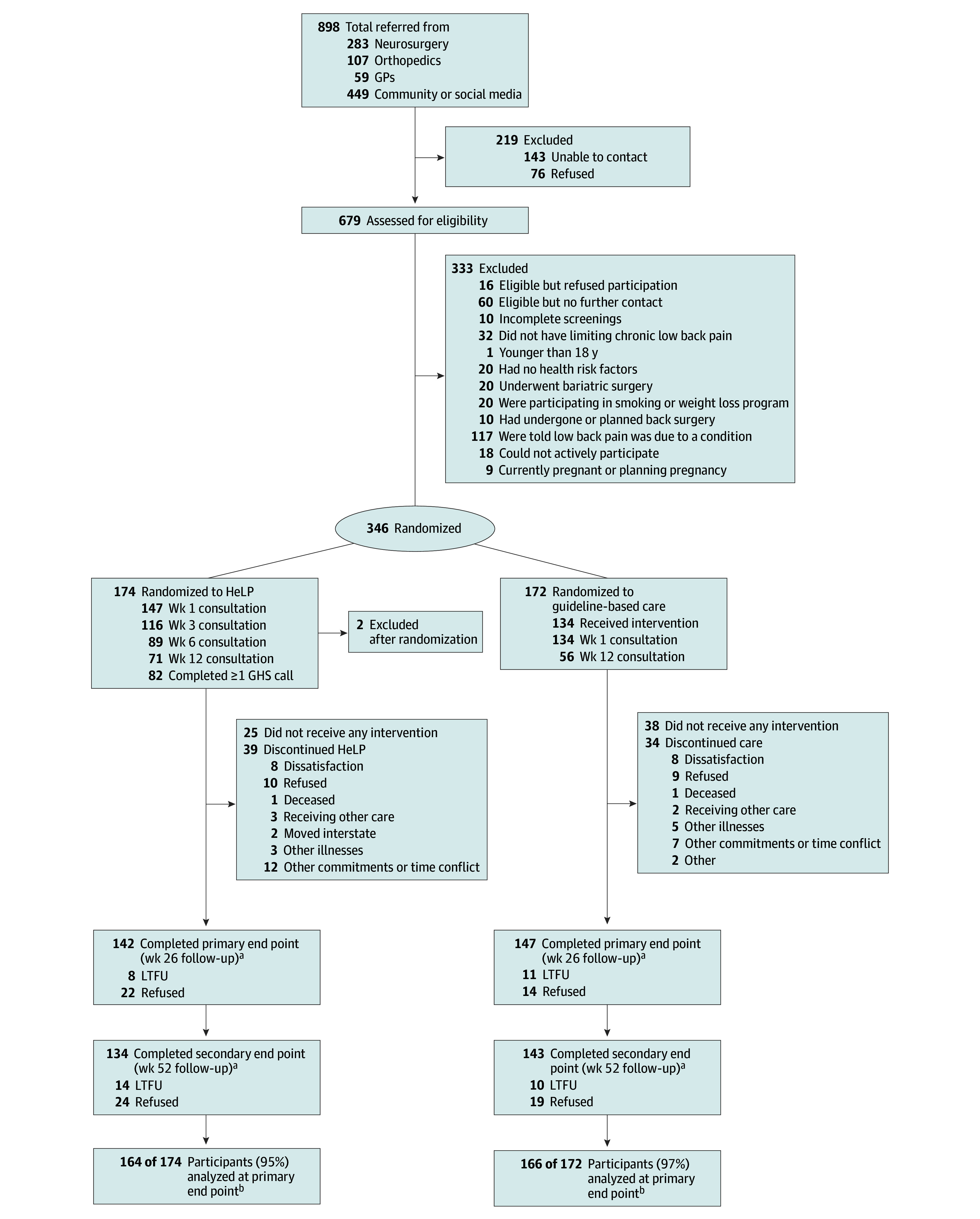
Patient Flow Diagram GHS indicates Get Healthy Service; GP, general practitioner; HeLP, Healthy Lifestyle Program; and LTFU, lost to follow-up. ^a^Some participants who did not complete all parts of the intervention still provided data at follow-up. ^b^Number analyzed is all participants included in primary outcome analyses in the intention-to-treat model and all participants with baseline Roland Morris Disability Questionnaire score and data for at least 1 follow-up time point.

Between September 2017 and November 2019, 679 patients (50% from hospital outpatients, 11% from general practitioners, and 39% from social media) were screened for eligibility (Figure 2). We excluded 247 ineligible patients, 76 were eligible but refused or were no longer contactable, and 10 did not complete screening. We randomized 346 eligible patients. Two participants from the HelP group were excluded after randomization due to ineligibility, subsequently revealed at the initial consultation, and were not included in analyses (1 participating in a weight loss study, 1 with planned surgery). The sample were 190 females (55%) and 154 males (45%) with a mean (SD) age of 50.2 (14.4) years, and due to randomization block sizes, 174 participants were allocated to the HeLP intervention and 172 to guideline-based care (control). Table 1 shows baseline characteristics.

**Table 1.** Baseline Characteristics

Characteristic	Patients, No. (%)	
	HeLP (n = 172)	Guideline-based care (n = 172)
Age, mean (SD), y	50 (13)	51 (15)
Sex		
Female	102 (59)	88 (51)
Male	70 (41)	84 (49)
Employment status, No./total No. (%)		
Employed or self-employed^a^	81/172 (47)	73/171 (43)
Cannot work due to health reasons	35/172 (20)	32/171 (19)
Unemployed	14/172 (8)	19/171 (11)
Home duties	10/172 (6)	5/171 (3)
Student	5/172 (3)	3/171 (2)
Retired	24/172 (14)	34/171 (20)
Other	3/172 (2)	4/171 (2)
Income, $ ($A)/y^b^		
Negative or none	3 (2)	0
≤33 799	61 (35)	65 (38)
33 800-88 399	75 (44)	68 (40)
88 400-207 999	22 (13)	29 (17)
≥208 000	1 (1)	1 (1)
Do not know	10 (6)	9 (5)
Private health insurance	45 (26)	50 (29)
Back pain duration, median (IQR), y	7.5 (3.0-17.0)	10.0 (4.0-20.0)
Episodes of back pain that have recovered, mean (SD) No.	3.1 (12.5)	2.0 (9.2)
Back pain compensable	10 (6)	12 (7)
Back pain with leg involvement	131 (76)	132 (77)
Have coexisting medical condition	130 (76)	119 (69)
Disability RMDQ score (range, 0-24), mean (SD)	14.7 (5.4)	14.0 (5.5)
Pain scale score (range, 0-10), mean (SD)	6.6 (1.7)	6.2 (1.8)
Weight, mean (SD)		
Self-reported, kg	90.3 (21.9)	94.0 (23.4)
Objectively measured, kg	92.4 (23.8)	96.5 (24.0)
BMI	31.4 (7.7)	32.5 (8.0)
Quality of life, mean (SD)		
Physical component score	48.8 (9.3)	49.2 (9.2)
Mental component score	50.0 (10.2)	49.9 (9.7)
Smoker, yes	46 (27)	42 (24)
>10 Cigarettes/d, smokers only^c^	22 (48)	19 (45)
Physical activity levels^d^		
METs, min/wk, mean (SD)	972 (1412)	1258 (2189)
Low, No./total No. (%)	99/171 (58)	95/170 (56)
Moderate, No./total No. (%)	50/171 (29)	53/170 (31)
High, No./total No. (%)	22/171 (13)	22/170 (13)
Nutrition Diet Quality score (range, 5-15), mean (SD)	10.5 (1.6)	10.4 (1.4)
No. of risk factors present^e^		
1	12 (7)	6 (4)
2	58 (34)	67 (39)
3	83 (48)	82 (47)
4	19 (11)	17 (10)
Poor sleep quality, yes	121 (70)	117 (68)
Pain self-efficacy score (range, 0-12), mean (SD)	7.5 (3.2)	7.8 (3.1)
Psychological distress score (range, 6-30), mean (SD)	14.4 (6.1)	14.1 (5.7)
Risky alcohol consumption	43 (25)	35 (20)
Using medication for back pain	150 (87)	146 (85)
Sought other care for back pain	101 (59)	95 (56)
Using community or carer support for back pain, No./total No. (%)	63/147 (43)	52/147 (35)
Days off work due to back pain (last 6 wk), median (IQR)	0.0 (0.0-2.0)	0.0 (0.0-1.5)
Days at work ill due to back pain (last 6 wk), median (IQR)	6.5 (3.0-18.0)	12.0 (2.0-24.5)

Abbreviations: BMI, body mass index (calculated as weight in kilograms divided by height in meters squared); HeLP, Healthy Lifestyle Program; MET, metabolic equivalent of task; RMDQ, Roland Morris Disability Questionnaire.

^a^ Full time, part time, casual, self-employed; denominator is 171 for guideline-based care group as there are missing data for 1 person for employment.

^b^ The conversion from Australian dollars to US dollars based on the exchange rate as of November 15, 2024, was A$1 = $1.55.

^c^ Denominator is the number of smokers.

^d^ Denominator is 171 for HeLP group and 170 for the guideline-based care group for physical activity categories (low, moderate, high) as there were missing data.

^e^ Risk factors (overweight, smoking, inadequate physical activity, inadequate intake of fruit and vegetable servings per day).

Follow-up data were provided by 164 of 172 participants (95%) in the HeLP group and 166 of 172 participants (97%) in the control group and were included in the intention-to-treat analysis (Figure 2). At the primary end point, 142 participants (83%) from the HeLP group and 147 partcipants (85%) from the guideline-based care group provided data. Participants without 26 weeks of data had lower baseline pain self-efficacy score (data missing: mean [SD] score, 4.9 [3.3]; data complete: mean [SD] score, 7.7 [3.2]). The most common reasons for discontinuing treatments were lack of time or other competing commitments (HeLP, 12 of 39 participants; guideline care, 7 of 34 participants) and dissatisfaction with the program (HeLP, 8 of 39 participants; guideline care, 8 of 34 participants).

### Intervention Delivery

HeLP participants attended a mean (SD) of 3.2 (1.9) consultations, while control participants attended a mean (SD) of 1.8 (1.4) consultations (eTable 2 in Supplement 2). Of the HeLP participants 147 of 172 (85%) accepted a referral to the Get Healthy Service; 82 of 147 (56%) commenced coaching calls, completing a median of 3 calls (IQR, 1-8 calls). Checklists and logs of intervention clinicians showed that more than 90% of all specified consultation components were delivered (eTable 3 in Supplement 2).

### Primary Outcome

Over 26 weeks, we observed a −1.3-point difference (95% CI, −2.5- to −0.2-point difference; *P* = .03) in disability favoring the HeLP intervention (Table 2). There was no evidence of a subgroup (moderation) effect for disability between participants with a healthy BMI and those with a high BMI (eTable 4 in Supplement 2).

**Table 2.** Effect on Primary, Secondary, Exploratory, and Safety Outcomes

Outcome, time point	HeLP (n = 172)	Guideline-based care (n = 172)	Mean difference (95% CI)^a^	*P* value
**Disability RMDQ score (range, 0-24), mean (SD)**				
Baseline	14.7 (5.4) [n = 172]	14.0 (5.5) [n = 172]	NA	NA
Week 6	12.5 (6.0) [n = 133]	13.3 (6.0) [n = 154]	−1.6 (−2.4 to −0.7)	<.001
Week 12	12.0 (6.2) [n = 129]	12.8 (6.4) [n = 142]	−1.6 (−2.6 to −0.5)	.004
Week 26	11.4 (6.5) [n = 142]	12.5 (6.7) [n = 147]	−1.3 (−2.5 to −0.2)	.03
Week 52	11.2 (7.0) [n = 134]	11.8 (7.2) [n = 143]	−1.0 (−2.2 to 0.3)	.12
**Pain intensity score (range, 0-10), mean (SD)**				
Baseline	6.6 (1.7) [n = 170]	6.2 (1.8) [n = 172]	NA	NA
Week 6	6.0 (2.2) [n = 131]	6.2 (2.0) [n = 147]	−0.4 (−0.8 to 0.0)	.07
Week 12	5.6 (2.5) [n = 122]	5.8 (2.4) [n = 137]	−0.4 (−0.9 to 0.1)	.09
Week 26	5.4 (2.5) [n = 130]	5.6 (2.3) [n = 133]	−0.3 (−0.8 to 0.2)	.24
Week 52	5.4 (2.6) [n = 129]	5.7 (2.5) [n = 132]	−0.5 (−1.0 to 0.1)	.09
**Weight, self-reported, mean (SD), kg**				
Baseline	90.3 (21.9) [n = 172]	94.0 (23.4) [n = 172]	NA	NA
Week 6	91.0 (23.7) [n = 130]	94.5 (23.6) [n = 142]	−0.1 (−1.3 to 1.2)	.94
Week 12	89.7 (23.6) [n = 120]	94.5 (22.5) [n = 130]	−1.1 (−2.3 to 0.0)	.06
Week 26	90.2 (23.1) [n = 128]	95.8 (24.2) [n = 131]	−1.6 (−3.2 to −0.0)	.049
Week 52	90.7 (22.7) [n = 126]	96.1 (22.9) [n = 128]	−0.7 (−2.2 to 0.8)	.36
**Weight, objectively measured, mean (SD), kg**				
Week 1	92.4 (23.8) [n = 140]	96.5 (24.0) [n = 131]	NA	NA
Week 12	94.0 (22.2) [n = 65]	95.4 (23.8) [n = 55]	−0.9 (−1.9 to 0.1)	.09
**BMI (self-reported height and weight), mean (SD)**				
Baseline	31.4 (7.7) [n = 172]	32.5 (7.9) [n = 172]	NA	NA
Week 6	32.0 (8.5) [n = 130]	32.9 (8.2) [n = 142]	−0.0 (−0.5 to 0.5)	.91
Week 12	31.6 (8.4) [n = 120]	32.8 (7.8) [n = 130]	−0.4 (−0.8 to 0.0)	.05
Week 26	31.4 (8.3) [n = 128]	33.1 (8.4) [n = 131]	−0.6 (−1.1 to −0.0)	.049
Week 52	31.6 (8.0) [n = 126]	33.3 (7.8) [n = 128]	−0.3 (−0.8 to 0.3)	.31
**Quality of life: physical component score (range, 0-100), mean (SD)**				
Baseline	48.8 (9.3) [n = 171]	49.2 (9.2) [n = 171]	NA	NA
Week 6	51.0 (10.3) [n = 132]	50.0 (9.9) [n = 151]	1.6 (0.2 to 3.1)	.03
Week 12	51.6 (10.2) [n = 126]	51.0 (10.0) [n = 140]	2.0 (0.4 to 3.6)	.02
Week 26	52.7 (10.5) [n = 138]	51.2 (10.7) [n = 140]	1.8 (0.1 to 3.4)	.04
Week 52	52.5 (11.0) [n = 131]	52.4 (11.2) [n = 139]	0.9 (−1.0 to 2.9)	.34
**Quality of life: mental component score (range, 0-100), mean (SD)**				
Baseline	50.0 (10.2) [n = 171]	49.9 (9.7) [n = 171]	NA	NA
Week 6	50.1 (10.0) [n = 130]	48.9 (9.2) [n = 151]	1.3 (−0.2 to 2.9)	.10
Week 12	50.5 (10.1) [n = 126]	49.7 (9.9) [n = 140]	1.5 (−0.3 to 3.3)	.11
Week 26	51.6 (10.5) [n = 138]	50.5 (9.8) [n = 141]	1.2 (−0.6 to 3.1)	.19
Week 52	50.4 (10.2) [n = 134]	50.2 (9.7) [n = 138]	0.6 (−1.4 to 2.6)	.55
**Smoker, No./total No. (%)**				
Baseline	46/172 (27)	42/172 (24)	NA	NA
Week 6	29/132 (22)	33/145 (23)	OR, 1.10 (0.25 to 4.84)	.90
Week 12	27/121 (22)	27/137 (20)	OR, 1.80 (0.38 to 8.54)	.46
Week 26	27/129 (21)	29/132 (22)	OR, 0.96 (0.20 to 4.52)	.96
Week 52	33/129 (26)	29/132 (22)	OR, 1.96 (0.43 to 8.98)	.39
**Smoke >10 cigarettes/d, No./total No. (%)^b^**				
Baseline	22/53 (42)	19/44 (43)	NA	NA
Week 6	14/35 (40)	12/37 (32)	OR, 1.37 (0.27 to 6.98)	.71
Week 12	14/35 (40)	8/31 (26)	OR, 3.31 (0.54 to 20.29)	.19
Week 26	11/35 (31)	8/33 (24)	OR, 1.30 (0.22 to 7.99)	.77
Week 52	14/37 (38)	9/32 (28)	OR, 2.20 (0.53 to 11.58)	.43
**Physical activity, mean (SD), METs min/wk**				
Baseline	972 (1412) [n = 171]	1257 (2189) [n = 170]	NA	NA
Week 6	1662 (1956) [n = 113]	2293 (3501) [n = 128]	−287 (−986 to 412)	.42
Week 12	1759 (1999) [n = 106]	1708 (2134) [n = 117]	285 (−216 to 787)	.26
Week 26	2031 (2622) [n = 109]	2116 (2674) [n = 118]	−19.3 (−623 to 584)	.95
Week 52	1989 (2453) [n = 119]	2196 (2939) [n = 118]	−89.5 (−746 to 567)	.79
**At least moderate activity levels, No./total No. (%)**				
Baseline	72/171 (42)	75/170 (44)	NA	NA
Week 6	69/114 (61)	82/132 (62)	OR, 0.95 (0.50 to 1.81)	.88
Week 12	71/110 (65)	70/120 (58)	OR, 1.38 (0.71 to 2.69)	.35
Week 26	74/113 (65)	75/121 (62)	OR, 1.13 (0.58 to 2.21)	.72
Week 52	76/121 (63)	78/122 (64)	OR, 0.98 (0.51 to 1.89)	.95
**Nutrition diet quality score (range, 5-15), mean (SD)**				
Baseline	10.5 (1.6) [n = 172]	10.4 (1.4) [n = 172]	NA	NA
Week 6	10.8 (1.5) [n = 129]	10.4 (1.4) [n = 145]	0.3 (−0.0 to 0.6)	.07
Week 12	10.9 (1.6) [n = 119]	10.4 (1.5) [n = 136]	0.4 (0.1 to 0.7)	.02
Week 26	10.8 (1.5) [n = 126]	10.4 (1.5) [n = 129]	0.3 (−0.0 to 0.7)	.08
Week 52	10.5 (1.6) [n = 129]	10.4 (1.6) [n = 131]	0.1 (−0.2 to 0.5)	.47
**Poor sleep quality, No./total No. (%)**				
Baseline	121/172 (70)	117/172 (68)	NA	NA
Week 6	70/131 (53)	83/147 (56)	OR, 0.81 (0.42 to 1.56)	.52
Week 12	67/121 (55)	76/136 (56)	OR, 0.90 (0.45 to 1.79)	.76
Week 26	69/130 (53)	67/131 (51)	OR, 1.12 (0.57 to 2.20)	.75
Week 52	61/129 (47)	73/132 (55)	OR, 0.60 (0.31 to 1.18)	.14
**Pain self-efficacy score (range, 0-12), mean (SD)**				
Baseline	7.4 (3.2) [n = 172]	7.8 (3.1) [n = 172]	NA	NA
Week 6	7.7 (3.3) [n = 131]	7.4 (3.3) [n = 147]	0.7 (0.1 to 1.3)	.02
Week 12	8.1 (3.1) [n = 121]	7.6 (3.3) [n = 136]	0.9 (0.3 to 1.6)	.005
Week 26	8.0 (3.4) [n = 129]	7.9 (3.1) [n = 130]	0.5 (−0.1 to 1.1)	.11
Week 52	8.1 (3.3) [n = 129]	7.8 (3.4) [n = 132]	0.9 (0.2 to 1.5)	.01
**Psychological distress (range, 6-30), mean (SD) score**				
Baseline	14.3 (6.0) [n = 172]	14.1 (5.7) [n = 172]	NA	NA
Week 6	14.1 (6.3) [n = 129]	14.2 (5.5) [n = 146]	−0.2 (−1.2 to 0.8)	.65
Week 12	13.6 (5.9) [n = 120]	14.0 (5.9) [n = 134]	−1.0 (−2.0 to −0.0)	.045
Week 26	13.4 (6.6) [n = 127]	14.1 (6.1) [n = 131]	−0.9 (−2.0 to 0.2)	.10
Week 52	13.0 (6.2) [n = 129]	14.1 (6.2) [n = 130]	−1.6 (−2.8 to −0.4)	.008
**Risky alcohol consumption, No./total No. (%)**				
Baseline	43/172 (25)	35/172 (20)	NA	NA
Week 6	27/130 (21)	26/143 (18)	OR, 1.03 (0.39 to 2.68)	.95
Week 12	20/120 (17)	23/136 (17)	OR, 0.70 (0.25 to 2.00)	.51
Week 26	22/128 (17)	20/128 (16)	OR, 0.97 (0.34 to 2.76)	.95
Week 52	25/129 (19)	21/130 (16)	OR, 1.04 (0.37 to 2.89)	.94
**Have coexisting medical condition, No./total No. (%)**				
Baseline	130/172 (76)	119/172 (69)	NA	NA
Week 6	95/138 (69)	101/158 (64)	OR, 1.18 (0.59 to 2.38)	.63
Week 12	90/139 (65)	99/148 (67)	OR, 0.64 (0.32 to 1.30)	.22
Week 26	84/147 (57)	91/150 (61)	OR, 0.69 (0.36 to 1.34)	.28
Week 52	89/137 (65)	98/148 (66)	OR, 0.76 (0.38 to 1.54)	.45
**Use medication for back pain, No./total No. (%)**				
Baseline	150/172 (87)	146/172 (85)	NA	NA
Week 6	79/131 (60)	91/147 (62)	OR, 0.83 (0.38 to 1.80)	.64
Week 12	81/121 (67)	86/135 (64)	OR, 1.08 (0.47 to 2.47)	.85
Week 26	83/130 (64)	84/131 (64)	OR, 1.02 (0.45 to 2.28)	.97
Week 52	72/128 (56)	85/131 (65)	OR, 0.57 (0.26 to 1.26)	.17
**Use health care services for back pain, No./total No. (%)**				
Baseline	101/172 (59)	95/171 (56)	NA	NA
Week 6	65/130 (50)	66/146 (45)	OR, 1.22 (0.67 to 2.23)	.51
Week 12	58/122 (48)	43/136 (32)	OR, 2.05 (1.08 to 3.86)	.03
Week 26	59/130 (45)	35/131 (27)	OR, 2.46 (1.30 to 4.67)	.006
Week 52	48/128 (38)	44/132 (33)	OR, 1.15 (0.61 to 2.17)	.67
**Using community or carer support for back pain, No./total No. (%)**				
Baseline	63/147 (43)	52/147 (35)	NA	NA
Week 6	44/118 (37)	44/133 (33)	OR, 1.19 (0.56 to 2.52)	.65
Week 12	34/109 (31)	31/124 (25)	OR, 1.12 (0.49 to 2.57)	.79
Week 26	37/119 (31)	25/120 (21)	OR, 1.52 (0.66 to 3.49)	.32
Week 52	41/129 (32)	32/131 (24)	OR, 1.11 (0.48 to 2.55)	.81
**No. of days off work due to back pain in last 6 wk, mean (SD)**				
Baseline	1.4 (2.3) [n = 82]	1.4 (3.8) [n = 76]	NA	NA
Week 6	2.3 (6.0) [n = 67]	1.2 (2.5) [n = 71]	0.4 (−0.5 to 1.3)	.38
Week 12	1.7 (6.0) [n = 59]	1.2 (3.5) [n = 64]	−0.4 (−1.7 to 0.9)	.52
Week 26	2.0 (6.7) [n = 67]	1.5 (5.0) [n = 66]	−0.9 (−2.4 to 0.6)	.24
Week 52	1.3 (4.3) [n = 63]	1.2 (4.2) [n = 60]	−0.7 (−2.6 to 1.3)	.51
**No. of days at work ill with back pain in last 6 wk, mean (SD)**				
Baseline	10.9 (10.4) [n = 82]	14.0 (11.8) [n = 76]	NA	NA
Week 6	8.9 (11.4) [n = 65]	9.7 (11.2) [n = 72]	−0.0 (−4.2 to 4.1)	.99
Week 12	8.1 (10.8) [n = 59]	10.2 (13.8) [n = 63]	−1.8 (−6.7 to 3.0)	.46
Week 26	9.3 (12.3) [n = 64]	7.3 (12.4) [n = 65]	2.3 (−2.5 to 7.0)	.35
Week 52	6.1 (10.0) [n = 61]	6.9 (9.8) [n = 59]	−2.2 (−6.6 to 2.3)	.34
**Adverse events, No./total No. (%)**				
Week 6	22/123 (18)	22/142 (15)	NA	.62
Week 12	18/109 (17)	21/123 (17)	NA	≥.99
Week 26	31/126 (25)	24/131 (18)	NA	.23
Week 52	26/128 (20)	23/129 (18)	NA	.07

Abbreviations: BMI, body mass index (calculated as weight in kilograms divided by height in meters squared); HeLP, Healthy Lifestyle Program; MET, metabolic equivalent of task; NA, not applicable; OR, odds ratio; RMDQ, Roland Morris Disability Questionnaire.

^a^ Mean differences are effect estimates of intervention minus control (95% CI) or ORs (95% CI). See eTables 6 to 10 in Supplement 2 for medications, health services, carer services tasks, and other illnesses reported.

^b^ All participants who stated that they smoked at any time point were included in the analysis to account for the participants who took up or quit smoking.

### Sensitivity Analyses of Primary Outcome

CACE analyses indicated a larger difference in disability (−5.4 points; 95% CI, −9.7 to −1.2 points; *P* = .01) among compliers favoring the HeLP intervention at 26 weeks, compared with “would-be compliers” in the guideline care group (Table 3). Results of other sensitivity analyses were similar to those of the primary analyses for the primary outcome (eTables 5 to 7 in Supplement 2).

**Table 3.** CACE Analyses

Outcome, time point	No.	CACE estimate (95% CI)^a^	*P* value
**Disability RMDQ score (range, 0-24)**			
Week 6	336	−6.3 (−9.6 to −2.9)	<.001
Week 12	282	−6.3 (−10.3 to −2.3)	.002
Week 26	284	−5.4 (−9.7 to −1.2)	.01
Week 52	272	−3.9 (−8.5 to 0.8)	.10
**Pain intensity score (range, 0-10)**			
Week 6	273	−1.7 (−3.2 to −0.1)	.03
Week 12	254	−1.8 (−3.6 to −0.0)	.045
Week 26	258	−1.4 (−3.3 to 0.5)	.14
Week 52	256	−2.0 (−4.0 to −0.0)	.048
**Weight, self-reported, kg**			
Week 6	267	0.1 (−4.6 to 4.8)	.97
Week 12	245	−3.8 (−7.9 to 0.3)	.07
Week 26	254	−5.5 (−11.3 to 0.4)	.07
Week 52	249	−2.1 (−7.6 to 3.4)	.45
**Quality of life: physical component score (range, 0-100)**			
Week 6	278	6.3 (0.8 to 11.8)	.02
Week 12	261	7.5 (1.4 to 13.6)	.02
Week 26	273	6.8 (0.7 to 13.0)	.03
Week 52	265	3.7 (−3.5 to 10.8)	.31
**Quality of life: mental component score (range, 0-100)**			
Week 6	281	5.3 (−0.9 to 11.6)	.09
Week 12	263	5.0 (−2.3 to 12.2)	.18
Week 26	279	4.6 (−2.7 to 11.9)	.22
Week 52	272	1.9 (−6.2 to 10.0)	.65
**Smoker**			
Week 6	272	OR, 0.85 (0.01 to 74.83)	.94
Week 12	253	OR, 2.21 (0.02 to 246.83)	.74
Week 26	256	OR, 0.65 (0.01 to 70.48)	.86
Week 52	256	OR, 2.81 (0.03 to 279.87)	.66

Abbreviations: CACE, complier average causal effect; OR, odds ratio; RMDQ, Roland Morris Disability Questionnaire.

^a^ CACE estimate is the mean difference of intervention minus control among compliers (95% CI) or OR (95% CI).

### Secondary and Exploratory Outcomes

At 26 weeks, there was a difference in weight assessment (−1.6 kg; 95% CI, −3.2 to −0.0 kg; *P* = .049) and physical functioning quality of life score (1.8; 95% CI, 0.1-3.4; *P* = .04) favoring the HeLP intervention (Table 2). Effect estimates for weight and physical functioning quality of life at other time points favored the HeLP intervention; however, 95% CIs crossed zero. There were no between-group differences in pain intensity score (−0.3; 95% CI, −0.8 to 0.2; *P* = .24), smoking status (odds ratio, 0.96; 95% CI, 0.20-4.52; *P* = .96), or mental functioning quality of life score (1.2; 95% CI, −0.6 to 3.1; *P* = .19) at 26 weeks. CACE analyses revealed larger but uncertain effects in most secondary outcomes, and a larger meaningful difference in physical functioning quality of life score (6.8; 95% CI, 0.7-13.0; *P* = .03) favoring HeLP (Table 3). Most exploratory outcomes had no meaningful differences between groups (Table 2; eTables 8-11 in Supplement 2).

### Adverse Events

There was no significant difference in adverse events between groups (HeLP group, 31 of 126 [25%]; control group, 24 of 131 [18%]; *P* = .23). Over the study period, 64 participants from the HeLP group and 70 from the guideline care group reported an adverse event, including exacerbation of a current condition (Table 2) and there were 2 unrelated deaths (1 in each group). *International Statistical Classification of Diseases and Related Health Problems, Tenth Revision* classifications and illnesses requiring health care are reported in eTables 12 and 13 in Supplement 2.

## Discussion

In this pragmatic randomized clinical trial, integrating healthy lifestyle into care for low back pain resulted in a small improvement in disability at 26 weeks compared with current guideline-based care alone. There was also a small reduction in weight and improved physical quality of life, but no differences in pain intensity, mental quality of life, or smoking. CACE analyses showed large clinically meaningful benefits of integrated lifestyle caare for disability, pain intensity, and physical function among compliers in the HeLP group, compared with guideline care.

Our trial provides high-quality evidence on the effectiveness of targeting lifestyle risks in the management of low back pain. Currently, clinical practice guidelines for low back pain do not recommend lifestyle-focused treatment approaches due to very-low-certainty evidence of benefit.^11,16^ Although effects in intention-to-treat analyses of our trial would be considered small, the disability improvements are over and above those seen with current best practice guideline care, suggesting that targeting lifestyle is of additional benefit to current recommended care.

Integrating clinical and preventive care services for people with chronic low back pain may have broader health benefit to individuals. A large proportion of patients with chronic low back pain have co-occurring unhealthy lifestyles,^35,36^ which increases their risk of developing other chronic diseases.^7^ We observed higher weight loss in the HeLP intervention group; however, the longer-term benefits and the effects of this approach on other lifestyle risks (eg, smoking, physical inactivity, and alcohol use) are uncertain. Our results show potential for how a condition-specific approach to integrated care can directly affect back pain outcomes as well as provide an opportunity for preventive care of other burdensome chronic diseases in an at-risk population group.

Our prespecified CACE analyses revealed that people who complied with approximately half of the HeLP intervention (2 consultations and 5 telephone calls) achieved clinically meaningful improvements in disability, pain, weight, and physical quality-of-life outcomes compared with those who received guideline-based care. Although it is uncertain whether compliance traits are modifiable, and whether improving compliance would lead to greater effects in “would-be” noncompliers, CACE estimates provide a more accurate indication of the effect of receiving the intended treatment. The results provide reassurance to health care payers, who incur the additional costs of providing treatment, that integrated lifestyle care yields meaningful benefit for those who receive and adhere with care.

HeLP was a complex model of care with multiple components and delivery mechanisms. Although the intervention was developed and planned as a pragmatic model of care, implementing integrated lifestyle care as tested in our trial may require additional resources and training in many jurisdictions. Engaging patients with coexisting back pain and lifestyle risks in integrated care interventions remains a challenge that may undermine extensive implementation. The benefits of integrating lifestyle care into low back pain management should be considered with respect to the complexity of treatment, costs, and an individual’s likely adherence.

Given the lack of evidence on lifestyle-focused care for low back pain management, more high-quality research is needed. Future studies should seek to understand the comparative effects of varying ways of providing lifestyle support, as well as the resources clinicians require to integrate these approaches into routine practice. Investigating the long-term benefits and sustainability of integrated treatment models for back pain, particularly related to future chronic disease risks, is warranted. Furthermore, exploring the use of digital technologies to deliver and support integrated lifestyle interventions may offer opportunities to enhance accessibility and engagement. Our planned cost-effectiveness evaluation, mediation analyses, and process evaluation aim to provide further insight about implementation and future opportunities to adapt healthy lifestyle approaches for low back pain management.

### Strengths and Limitations

The study has several strengths. The HeLP trial was a large, multisite pragmatic randomized clinical trial. Previous trials of lifestyle interventions for low back pain have focused on weight loss, found inconsistent effects, used small sample sizes, and were limited by high risk of bias.^8,9,10,11^ Our trial followed prepublished protocols and statistical analysis plans and had low data attrition. Results of our sensitivity analyses provide greater confidence in the main findings, as they either closely approximated the findings of main analyses or suggested more meaningful benefit. Our recruitment was inclusive, taking patients from different care providers and the community, which are all common care access options for people with low back pain in economically developed countries.

The trial also had some limitations, including the inability to blind clinicians and participants to treatment groups. However, we limited performance bias by not informing participants of the specifics of treatment group details being tested and by having clinicians deliver care to either the intervention or control group, not both. The sample participants had a high proportion of patients deemed ineligible due to suspected serious causes for back pain (eg, cancer, rheumatoid arthritis, fracture; 17% vs population prevalence of approximately 1%), which may be a consequence of a large number of patients referred from secondary care.^37^ The sample participants also had a long duration of preceding back pain (median, 7.5-10 years), and we did not collect extensive data on racial or ethnic background of participants. These characteristics may limit the generalizability of our results to some populations. Finally, although adherence to treatment was at least equivalent to other studies of complex interventions,^9,10^ nearly one-fourth of participants discontinued care over the 6-month intervention period, and more participants in the intervention group had accessed alternative care at 26 weeks.

## Conclusions

This randomized clinical trial suggests that integrating management of healthy lifestyle risks for the care of patients with low back pain may provide a small improvement in disability compared with guideline-based care alone and large benefit for those who complied with at least half the treatment. Treatment models that include support to address lifestyle behaviors hold promise for improving the disability burden of low back pain and providing opportunistic care for prevalent chronic disease risks in those with back pain.

## Notes

Supplement 1.
Trial Protocol and Statistical Analysis Plan

Supplement 2. **eAppendix.** Inclusion and Exclusion Criteria **eTable 1.** Outcomes and Data Collection Timepoints **eTable 2.** Participant Fidelity (Adherence and Participation), Reasons for Withdrawal and Satisfaction With Care **eTable 3.** Clinician Reported Intervention Fidelity **eTable 4.** Moderation Analyses - Difference in Mean RMDQ Score Between Groups by BMI Category **eTable 5.** Characteristics of Compliers and Noncompliers for the HeLP Intervention **eTable 6.** Sensitivity Analysis - Difference in the Mean Imputed RMDQ Score Between Treatment Groups **eTable 7.** Sensitivity Analysis - Difference in the Mean RMDQ Score Between Treatment Groups Adjusted for Imbalanced Prognostic Variables **eTable 8.** Common Medications Classifications Used for Back Pain by Group **eTable 9.** Common Healthcare Services Used for Low Back Pain **eTable 10.** Carer or Community Services Support Used **eTable 11.** Activities Requiring Help With From Carers or Community Support **eTable 12.** Frequency of Adverse Events (*IDC10* Categories) by Group **eTable 13.** Other Illnesses Requiring Medication or Health Care

Supplement 3. **Nonauthor Collaborators.** Healthy Lifestyle Program for Chronic Low Back Pain (HeLP) Trial working group members

Supplement 4.
Data Sharing Statement
