# Supplementary material for: Healthy Lifestyle Care vs Guideline-Based Care for Low Back Pain: A Randomized Clinical Trial
Source: JAMA Netw Open. 2025 Jan 10;8(1):e2453807. doi: 10.1001/jamanetworkopen.2024.53807 (PMC11724347; doi:10.1001/jamanetworkopen.2024.53807)
Supplement: Supplement 1. — Trial Protocol and Statistical Analysis Plan [file jamanetwopen-e2453807-s001.pdf]

National Ethics Application synopsis for - HELP - A healthy lifestyle program for patients with chronic low back pain

This synopsis is reported in accordance with the SPIRIT statement.

Administrative Information

Title: *HELP - A healthy lifestyle program for patients with chronic low back pain*

Trial Registration

In preparation

Protocol Version

March 2017.

Funding

The project is fully funded by an NHMRC project grant (APP1100992) 2016-2020.

Roles and Responsibilities

Chief investigators

**Name**	**Dr Christopher Williams**
**Position:**	Clinical Research Fellow at Hunter New England Population Health
**Affiliations:**	Hunter New England Local Health District, University of Newcastle, The George Institute
**Address:**	Hunter New England Population Health, Locked Bag 10 Wallsend NSW 2287
**Phone:**	0249246374
**Roles:**	Dr Williams will oversee planning and implementation of the research project and intervention delivery. He will be responsible for study deliverables and reporting to required parties including NHMRC, and LHD and NSW Health partners.

**Name**	**Dr Steven Kamper**
**Position:**	Senior Research Fellow at The George Institute
**Affiliations:**	The George Institute, Hunter New England Local Health District
**Address:**	PO Box M201 Missenden Road Camperdown NSW 2050
**Phone:**	02 80524408
**Roles:**	Dr Kamper will support Dr Williams oversee planning and implementation of the research project and intervention delivery. He will support study deliverables and reporting to required parties

Research Team

**Name**	**Dr Hopin Lee**
**Position:**	Research Fellow
**Affiliations:**	Hunter New England Local Health District, University of Newcastle
**Address:**	Hunter New England Population Health, Locked Bag 10 Wallsend NSW 2287
**Phone:**	02 4924 6367
**Roles:**	Mr Lee will contribute to the planning, implementation, evaluation, and dissemination of the project. He will also provide input into the musculoskeletal management component of the intervention.

**Name**	**Ms Emma Robson**
**Position:**	Project Officer
**Affiliations:**	Hunter New England Local Health District, University of Newcastle
**Address:**	Hunter New England Population Health, Locked Bag 10 Wallsend NSW 2287
**Phone:**	02 4924 6069
**Roles:**	Ms Robson will contribute to the planning, implementation, evaluation and dissemination of the project.

**Name**	**Ms Priscilla Viana Silva**
**Position:**	PhD student
**Affiliations:**	Hunter New England Local Health District, University of Newcastle
**Address:**	Hunter New England Population Health, Locked Bag 10 Wallsend NSW 2287
**Phone:**	02 4924 6206
**Roles:**	Ms Silva will contribute to the planning, implementation, evaluation and dissemination of the project.

**Name**	**Mr Connor Gleadhill**
**Position:**	Research Assistant
**Affiliations:**	Hunter New England Local Health District, University of Sydney
**Address:**	Hunter New England Population Health, Locked Bag 10 Wallsend NSW 2287
**Phone:**	NA
**Roles:**	Connor will contribute to the planning, implementation, evaluation and dissemination of the project with expertise in physiotherapy.

**Name**	**Mr Simon Davidson**
**Position:**	PhD Student
**Affiliations:**	Hunter New England Local Health District, University of Sydney
**Address:**	Hunter New England Population Health, Locked Bag 10 Wallsend NSW 2287
**Phone:**	NA
**Roles:**	Simon will contribute to the planning, implementation, evaluation and dissemination of the project with expertise in physiotherapy.

**Name**	**Miss Lauren Devine**
**Position:**	Research and Communication Manager
**Affiliations:**	Hunter New England Local Health District, University of Newcastle
**Address:**	University of Newcastle, University Drive, Callaghan NSW 2308
**Phone:**	02 4924 6601

**Roles:**	Miss Divine will contribute to the planning, implementation, evaluation and dissemination of the project.

**Name**	**Miss Eliza Magennis**
**Position:**	Research Assistant
**Affiliations:**	Hunter New England Local Health District, University of Newcastle
**Address:**	University of Newcastle, University Drive, Callaghan NSW 2308
**Phone:**	
**Roles:**	Miss Magennis will contribute to data collection, monitoring, implementation, evaluation and dissemination of the project.

Introduction

Background and Rationale

Low back pain is common and responsible for huge societal burden, but contemporary management approaches that have been researched including analgesics, manual therapy and exercise, do not provide lasting benefit to patients. While numerous studies show associations between chronic low back pain and health behaviour risks such as smoking, overweight/obesity physical inactivity and poor diet, there have been no trials conducted to determine if interventions effective in reducing these risk factors, have an impact on low back pain.

Our research shows usual practice does not support low back pain patients reduce health behaviour risks. Instead low back pain patients are referred for surgical consultation where they commonly wait for extended periods of time (>18 months) during which time they receive no care and their symptoms and health deteriorate.

Pilot data collected by the team indicates:

- 70% of back pain patients waiting for surgery consultation are overweight, and 27% are current smokers, greater than 50% are inactive and nearly 90% report suboptimal diet.
- These patients are highly motivated to improve these health behaviours.
- Telephone-based support is the preferred mode of access to care, and taken up by 80% patients.
- This intervention delivery method is well-established and cost-effective.
- Patient engagement and recruitment methods are feasible.
- There is clinical and executive support for this intervention, at local and state levels.

A randomised controlled trial will be conducted to determine the effect of evidence-based advice and education, supplemented by a healthy lifestyle coordinated care program on low back pain related disability compared to usual care. The intervention has the potential to reduce the burden of low back pain, a condition hampered by ineffective treatments, by integrating several existing strategies which target health problems known to be associated with low back pain, but which have never been researched in a trial of this context.

The intervention is novel approach integrative of clinical care and telehealth, using methods shown to be effective, cost-effective and highly accepted by patients. While there is significant potential to impact low back pain outcomes, the approach is also likely to achieve health system benefits by reducing unnecessary care, and improving health by targeting risk factors for several other chronic

diseases. If effective the model or care may provide a template to cater for high referral volumes to public hospitals in related conditions.

Explanation for choice of comparators
The comparison group is usual care. Participants allocated to the control group will receive usual care physiotherapy. Further description of usual care is provided in the interventions section.

Objectives
The primary aim of the HELP study is to determine the effect of a coordinated healthy lifestyle program, involving evidence-based advice and education for low back pain, and telephone-based healthy lifestyle coaching, on disability in low back pain patients who have a health behaviour risk (overweight, smoker, inactive, poor diet).

Trial Design
The study will be a parallel, 1:1 cohort, superiority, blinded (patient, outcome assessor) randomised controlled trial of patients with chronic low back pain.

Methods: Participants, interventions and outcomes

Study Setting
The study will be conducted in the Hunter New England Local Health District at the Royal Newcastle Centre of the John Hunter Hospital, in conjunction with Hunter New England Population Health. Data will be collected in Australia only.

Eligibility Criteria
Inclusion criteria – Eligible patients will be: 18 years of age or older with chronic low back pain, defined as pain between the 12th rib and buttock crease, with or without leg pain of more than 3 months duration; have at least one health risk factor (overweight BMI >25kg/m^2, do not meet the recommendations for physical activity or fruit and vegetable consumption, smoker).
Exclusion criteria - Patients will be excluded if they: are unable to use a telephone; cannot actively engage in the intervention program; do not live independently (i.e. require care for activities of daily living); are currently undertaking weight management or smoking cessation programs; have planned surgery in the next 6 months; or have a comorbidity that precludes understanding or completion of study procedures.
Ineligible patients will be invited to register in a Musculoskeletal service cohort (ethics approval: 13/12/11/5.18). These people will be provided with an information letter as previously approved and contacted by the study team if they consent to this.

Interventions:
Intervention group
Patients randomised to the intervention group will receive an intervention with three components: a) assessment and delivery of in-depth advice and education by a physiotherapist regarding low back pain and proactive referral to b) a telephone-based weight management coaching (diet and exercise) program (*Get Healthy*) and c) a telephone-based smoking cessation counseling service (*Quitline*) for those participants who identify as smokers. Detail regarding the study intervention follows.

a) Low back pain assessment, advice and monitoring (week 1, 3, 6 and 12)

An experienced physiotherapist will conduct an initial clinical assessment and provide condition specific advice and education, along with formative instructions about the coordinated care intervention. The advice will incorporate international guideline recommended education and reassurance, with explanation of the mechanisms by which weight loss, physical activity, improving diet and smoking cessation can improve back pain symptoms, and reduce health risks. Specifically, provision of advice aims to improve understanding of self-management principles for low back pain, reinforce the need to stay active or gradually increase activity, and avoid bed rest,[1] along with a clinically delivered rationale for the treatment approaches. Subsequent appointments may be also be partly delivered by other trained Allied Health Professionals such as a dietitian to assist in lifestyle education.

b) Weight management, physical activity and diet coaching program – Get Healthy (week 1 to 12)

All patients will be referred to a 3-month telephone-based healthy lifestyle coaching program delivered by allied health professionals from the NSW *Get Healthy Service*. Each session aims to assist patients to set goals regarding change in diet and physical activity and to overcome barriers to making such changes. All content and advice is provided according to standardised protocols and aligns with national diet and physical activity guidelines

All sessions will be based on self-regulation principles, including self-monitoring, goal setting, cognitive restructuring, problem solving, and environmental management, to assist patients set goals, regarding the change in diet and physical activity and overcome barriers to making such changes. Get Healthy coaches will be provided with low back pain specific training to tailor content for study participants if required.

c) Smoking cessation program - Quitline (week 12)

Participants who smoke or report they have 'quit' in the last month, will be referred to the *NSW Quitline*. Quitline counsellors (advisors) who are trained in health/education/psychology and smoking cessation, will contact participants, and systematically record information on smoking history and previous quit attempts. Advisors encourage smokers to set a quit date, and assist in maximizing success of quitting by guiding participants through evidence-based cessation methods with consideration of individual preferences and past history of quitting. Advisors also assist callers to deal with issues such as withdrawal symptoms. After the initial contact, and establishment of a quit date, participants will be phoned on the following occasions for support: the day before the agreed quit date; within the first two days after the quit date; within the first week of the quit date; twice within the following three weeks; three months after the quit date.

After the first call, participants will also be sent a 'Quit Kit' consisting of: i) 'Quit because you can' booklet, which includes a step-by-step guide to quitting; ii) 'Products to help you Quit smoking' guide, which outlines products known to aid in quitting, such as bupropion tablets, varenicline tablets, and nicotine replacement therapies (e.g. patches, gum, lozenges, tablets and inhalers; iii) 'You can Quit' pocket guide that contains simple messages and hints on how participants can stay a non-smoker; iv) a 'Choosing the best way to Quit' booklet to guide participants through the options for assistance and medications available to help them quit and help determine which is best suited for them.

The primary physiotherapist delivering assessment, advice, and monitoring will act as coordinated care liaison for The Get Healthy Service and Quitline programs. These strategies are aimed to enhance uptake and adherence.

The intervention will be discontinued if adverse events of unexpected type, severity, or frequency

are encountered. The intervention will also be discontinued at the request of Hunter New England HREC after evaluation of reported adverse events.

There will be no restrictions on concomitant care or prohibited interventions during the trial.

Control group:
Patients allocated to the control group will receive usual (guideline-based) management from the hospital physiotherapy department. No systematic process for the provision of lifestyle weight management or smoking cessation care to patients currently exists within the study hospital. Patients may receive care from their GP/surgeon, or access care independently. Receipt of any care will be measured at each follow up.

Outcomes

Primary
The primary outcome will be disability measured on the Roland Morris Disability Questionnaire - a 24 item low back pain specific survey, which has been extensively validated.

Secondary
- Pain – numerical pain rating scale
- Anthropometric data - weight change (kg), BMI, waist circumference (using ISAK protocols)[11]
- Sleep Quality (Item 6 Pittsburgh Sleep Quality Index)
- Quality of life (SF-12v2)
- Pain and self-efficacy (PSEQ-2)
- Psychological distress (Kessler 6)
- Smoking abstinence (NSW Health Survey)
- Physical activity and nutrition behaviours: International Physical activity Questionnaire, Short Food Frequency Questionnaire
- Alcohol consumption (AUDITC)
- Adverse events
- Patient Satisfaction
- Recommendation for surgery (for patients who progress to consultation)
- Smoking cessation and use of Quitline questions (intervention participants only)
- Mobile Applications Rating Scale (MARS) at 12 months only

Economic outcomes: Health care utilisation data extracted from patients self-reported health care, community care and medication utilisation. Intervention delivery cost data will include: staff time, telephone calls, written materials, referral costs, and stationery. Telephone–based program costs will be obtained from the services. Patient's absenteeism and presenteeism at work will also be collected.

Process Outcomes:
Patients (n=30, randomly selected) and clinicians involved in HeLP will be invited at the end of the study (12 month follow up) to participate in qualitative data capture via semi structured interviews. The interviews can be conducted face to face, or over the phone (based on personal preference) and aim to better understand patient and clinician attitudes and experience of the HeLP program and how to improve care for musculoskeletal conditions in the future.

Participant Timeline

Population: Low back pain patients referred to, or waiting for surgeon consultation at the Royal Newcastle Centre, with duration of pain longer than 3 months.

Central Randomisation (1:1 concealed n=346)
Permuted block approach to equally distribute overweight/obesity and smokers across groups

Healthy lifestyle intervention (n =173)
(1) Physio assessment and guideline advice
(2) Coordination of telephone programs:
- In-depth advice and education
- Weight loss, activity and diet (all)
- Smoking cessation (smokers or quit in last month)

Usual care (n =173)
(1) Physio assessment and guideline advice
(2) Usual care

Blind follow up:
Clinical follow up at 6 and 12 weeks; telephone follow up at 26 weeks and 12 months.
Primary outcome – back pain disability

Blind analyses:
Intention to treat
Linear mixed models - test of between group differences
Economic analysis – cost-utility analyses from societal and health sector perspectives

Sample Size

We calculated sample size using the method of Twisk for mixed models. With 3 repeated observations, an estimated intra-cluster correlation (correlation between the observations) of 0.5, alpha set at 5%, and allowing for up to 18% non-compliance, we require 346 patients to have an 80% power to detect between group differences of 3 points on Roland Morris Disability Questionnaire (SD 5) at 6 months. This is the smallest worthwhile effect that would justify implementation of the intervention. In these calculations we have conservatively ignored the increase in statistical power conferred by baseline covariates and stratification.

Recruitment

Patients who are:

a) Referred for orthopaedic or neurosurgery consultation at Outpatient Services, JHH for low back pain who do not require further consultation with a surgeon for their back pain or
b) Referred to the study directly by their treating clinician (i.e. GP, physiotherapist or surgeon) or,
c) Self-refer to the study by directly contacting the research team from social media/ community advertising; will all be provided with an information letter about the study, and a written consent form. The letter will advise that a Hunter New England employee will call them within the next 2 weeks to conduct a telephone survey and assess their eligibility for a new service being delivered for patients with low back pain. The letter also contains a toll-free number in the case that patients do not wish to be contacted, patients who do not wish to be contacted will not be called. During the phone call patients will be asked to provide verbal consent to participate and asked to fill in the written consent form (attached to the

information letter) and bring it with them to first clinical consultation of the study. Patients will be advised that they can withdraw consent at any time with no influence to the care they receive from the hospital. If eligible, patients in both groups will receive further clinical appointments at 6 and 12 weeks. Patients in the intervention group will be asked to attend an additional two clinical appointments (week 1, 3). Outcome assessment will be conducted on four subsequent occasions: week 6, 12 weeks, 26 weeks and 12 months post randomisation. The research team has successfully applied these procedures in the pilot research.

Methods Assignment of Interventions

Allocation:

Eligible consenting patients will be randomly allocated to the intervention or control group (1:1) after baseline assessment Randomisation will be disclosed to align with the usual practices of the hospital physiotherapists and reduce burden on clinical appointment times. Patients will be randomised using a central randomisation service to ensure concealment of treatment allocation. A permuted block randomization approach will be used so that the distribution of overweight and obesity and smokers across treatment conditions (intervention or control) will be maintained regardless of their prevalence in the final sample size.

Blinding:

It is proposed that the study will involve concealment to study group. There is no increased risk of harm, as both the intervention and the usual care group will receive the same usual care with the exception of the intervention group receiving additional advice and referral to Get Healthy Service and if a smoker, the Quitline. Blinding of participants to the intervention or usual care control group is proposed to reduce performance bias by participants should they be aware of treatment group.

All outcome assessors and investigators will also be blind to group allocation

Methods data collection, management, and analysis

Data collection Methods:

Follow up data will be collected by trained assessors via computer automated telephone interview (CATI). Patients will also be provided with reimbursement of their time and any associated costs to complete the follow up surveys. We will offer all patients yet to complete the 6 month follow up $60 (in the form of a Coles or Woolworths voucher).

Data management:

The integrity of trial data will be monitored by regularly scrutinising data files for omissions and errors. Electronic data will be stored on a secure server and paper copies located in a locked cabinet. Data will only be accessible by researchers and participant confidentiality will be maintained through secure data storage, during and post-trial.

Statistical methods:

Data will be analysed by a statistician who is blind to group status. Outcome analyses will be by intention-to-treat, with the number of analyses restricted and specified a priori in order to reduce the possibility of Type I errors. For the primary outcome, a P value of <0.05 will be considered statistically significant, while for the key secondary outcomes a P value of <0.025 will be considered significant. We will analyse the effect of treatment separately for each outcome using linear mixed models with random intercepts for individuals to account for correlation of repeated measures. We will obtain estimates of the effect of the intervention and 95% confidence intervals by constructing linear contrasts to compare the adjusted mean change (continuous variables) or difference in proportions (dichotomous variables) in outcome from baseline to each time point between the treatment and control groups. We will test the differential effect of the intervention based on

weight status (normal vs overweight) by including an interaction term (baseline weight status) in the primary model.

As we are investigating the effectiveness of a multi-component intervention it is important to understand the processes that lead to successful outcome, or not, as this will help refine future work. Chief Investigator 2 Kamper has developed skills in mediation analysis. We will investigate the mechanisms underlying our interventions using causal mediation analysis, to assess the mediating effect of physical activity, diet, pain self-efficacy, fear avoidance beliefs and symptoms of psychological distress (Depression Anxiety & Stress Scale (DASS 21) on low back pain disability.

Three economic analyses will be conducted. First, a cost model will be prepared comparing the costs associated with the control and intervention pathways. Second, a cost-utility analysis conducted from the perspective of the health sector. Third, a cost-utility analysis from a societal perspective in which the additional costs (and cost savings) associated with use of community services will be conducted. Intervention costs along with costs of other health care services will be factored in with levels of utilisation to estimate the costs of healthcare use for participants. Other health care services will be valued at standard rates published by the Australian Government. Private non-medical healthcare services will be valued at standard rates published by the relevant professional body or a third party payer. Costs of community services will be based on the self-reported costs to participants. Health state utilities, required to estimate quality-adjusted life-years, will be based on measures obtained from the SF-12 and transformed into health state utilities via the SF-6D algorithm.

Data Monitoring

The role of the data monitoring committee will be to handle and scrutinise all data collected from this trial. The committee is independent from the sponsor and has no competing interests.

Harms

No adverse events are anticipated however patients will be provided with a phone number of the Hunter New England HREC as an avenue for reporting adverse events should they arise. The details of any reported adverse events received by research staff will be documented according the HREC guidelines by the principal investigator as soon as possible. The project will be discontinued at any time at the request of the HREC after reviewing reported incidents. Ongoing reviews will be undertaken to ensure the project remains valuable in terms of the data provided to researchers and acceptability to patients. If for any reason the study is discontinued, patients will be informed that they will not be required to participate in data collection however they are able to continue participating in the GHS or Quitline programs outside of the trial.

The research team acknowledges that the program deals with potentially sensitive and personal issues and we have put measures in place to avoid and manage potential risks. For example, all data will be kept confidential and patients will be informed they can withdraw consent at any time. If any concerning information is disclosed by patients in consults (for example, mental health issues), permission will be sought from patients to contact their GP for follow up. Prior to enrolment, patients will be informed of any potential risks or discomfort by participating in the program. Participants will be monitored closely for adverse events. All adverse events will be recorded. Serious adverse events will be assessed by study investigators with clinical expertise and management appropriately.

Protocol amendments

If important protocol modifications need to be made, these will be reported to all partners, trial sites, trial registries, and journals.

Consent of Assent

Patients will be provided an information letter detailing the research (Appendix A), their participation, advising them they can withdraw at any time, the provision of a phone number to decline participation in the study and the research team contact details. Patients will be contacted 48 hours following provision of the information statement to confirm their eligibility, ask for verbal consent to participate in the study and book a face to face consult with a physiotherapist.

Confidentiality

All participants will be assigned a study ID code on inclusion into the study which will be provided to the Quitline service as part of the referral process. This identifying information is required to link individual outcome data collected as part of the study with health care utilisation data collected via the Quitline service. Once linked the data will only be stored with study IDs with names removed.

Paper copies of consent forms and questionnaires will be kept in secure storage and destroyed 5

years after completion of the study in accordance with NSW Health policy. Electronic files from the project will be stored indefinitely on password protected Population Health servers.

Declaration of Interests
None to declare.

Access to data
Only the Principal Investigators, Associate investigators, Research Assistants, and Physiotherapists named in the NEAF application will have access to information collected from participants. Should any additional research personnel join the team, an ethics variation will be submitted prior to them gaining access to such data.

Dissemination Policy
Study results will be disseminated in research reports (ie. to funding bodies), peer reviewed journal articles and conferences. No individual data will be identifiable in dissemination as only aggregate data will be presented.
