# Supplementary material for: Healthy Lifestyle Care vs Guideline-Based Care for Low Back Pain: A Randomized Clinical Trial
Source: JAMA Netw Open. 2025 Jan 10;8(1):e2453807. doi: 10.1001/jamanetworkopen.2024.53807 (PMC11724347; doi:10.1001/jamanetworkopen.2024.53807)
Supplement: Supplement 2. — eAppendix. Inclusion and Exclusion Criteria eTable 1. Outcomes and Data Collection Timepoints eTable 2. Participant Fidelity (Adherence and Participation), Reasons for Withdrawal and Satisfaction With Care eTable 3. Clinician Reported Intervention Fidelity eTable 4. Moderation Analyses - Difference in Mean RMDQ Score Between Groups by BMI Category eTable 5. Characteristics of Compliers and Noncompliers for the HeLP Intervention eTable 6. Sensitivity Analysis - Difference in the Mean Imputed RMDQ Score Between Treatment Groups eTable 7. Sensitivity Analysis - Difference in the Mean RMDQ Score Between Treatment Groups Adjusted for Imbalanced Prognostic Variables eTable 8. Common Medications Classifications Used for Back Pain by Group eTable 9. Common Healthcare Services Used for Low Back Pain eTable 10. Carer or Community Services Support Used eTable 11. Activities Requiring Help With From Carers or Community Support eTable 12. Frequency of Adverse Events (IDC10 Categories) by Group eTable 13. Other Illnesses Requiring Medication or Health Care [file jamanetwopen-e2453807-s002.pdf]

Supplementary Online Content

Mudd E, Davidson SRE, Kamper SJ, et al; Healthy Lifestyle Program (HeLP) for Chronic Low Back Pain Trial working group. Healthy lifestyle care vs guideline-based care for low back pain: a randomized clinical trial. *JAMA Netw Open.* 2025;8(1):e2453807. doi:10.1001/jamanetworkopen.2024.53807

eAppendix. Inclusion and Exclusion Criteria
eTable 1. Outcomes and Data Collection Timepoints
eTable 2. Participant Fidelity (Adherence and Participation), Reasons for Withdrawal and Satisfaction With Care
eTable 3. Clinician Reported Intervention Fidelity
eTable 4. Moderation Analyses - Difference in Mean RMDQ Score Between Groups by BMI Category
eTable 5. Characteristics of Compliers and Noncompliers for the HeLP Intervention
eTable 6. Sensitivity Analysis - Difference in the Mean Imputed RMDQ Score Between Treatment Groups
eTable 7. Sensitivity Analysis - Difference in the Mean RMDQ Score Between Treatment Groups Adjusted for Imbalanced Prognostic Variables
eTable 8. Common Medications Classifications Used for Back Pain by Group
eTable 9. Common Healthcare Services Used for Low Back Pain
eTable 10. Carer or Community Services Support Used
eTable 11. Activities Requiring Help With From Carers or Community Support
eTable 12. Frequency of Adverse Events (*IDC10* Categories) by Group
eTable 13. Other Illnesses Requiring Medication or Health Care

This supplementary material has been provided by the authors to give readers additional information about their work.

eAppendix. Inclusion and Exclusion Criteria

Adult patients ≥18 years of age, who met the following criteria were eligible:

- Primary complaint of chronic low back pain, defined as pain between the 12th rib and buttock crease with or without leg pain for a duration longer than 3 months since onset of pain;
- Average low back pain intensity ≥3 out of 10 over the past week, rated on a 0-10 numerical rating scale (NRS), OR at least a moderate level of interference with normal daily activities of daily living (adapted from item 8 on Short Form -36) over the last week;
- Had one or more of the following lifestyle risk factors: overweight ((Body Mass Index (BMI) >25)), current smoker, participation in less than 30 minutes of physical activity on at least 5 days of the week; consumes less than 2 serves of fruit and 5 serves of vegetables per day.

Patients were excluded if they:

- Previously had bariatric weight loss surgery;
- Were already undertaking a weight loss or smoking cessation program;
- Had back surgery in the previous 6 months or planned back surgery in the next 6 months;
- Had a known or suspected serious pathology causing back pain (i.e. rheumatoid arthritis, confirmed radiculopathy including both sensory and motor deficits, cancer, fracture or infection);
- Could not actively engage in the intervention (unable to communicate, use a telephone or attend appointments, or unable to adapt meals or exercise);
- Comorbidity that does not allow safe completion of study procedures (e.g. uncontrolled blood pressure or heart conditions, uncontrolled diabetes);
- Were pregnant or planning pregnancy in the next 12 months.

© 2025 Mudd E et al. *JAMA Network Open.*

eTable 1. Outcomes and Data Collection Timepoints

Construct	Measure	Time (week)
**Primary outcome**		
Disability	Roland Morris Disability Questionnaire (RMDQ).	0, 6, 12, 26, 52
**Secondary outcomes**		
Pain Intensity	11 point (0-10) Numerical Rating Scale, as the average pain over the last week where zero indicated no pain and ten indicates worst possible pain.	0, 6, 12, 26, 52
Weight	Objective weight measured to the nearest 0.1kg using International Society for the Advancement of Kinanthropometry (ISAK) procedures.	1, 12
	Self-reported weight (kg)	0, 6, 12, 26, 52
Quality of Life	12-item Short Form Health Survey version 2 (0-100 scale, higher score indicates greater quality of life).	0, 6, 12, 26, 52
Smoking Status	Current smoking status and how many cigarettes smoked per day; from the NSW Population Health Survey.	0, 6, 12, 26, 52
**Exploratory outcomes**		
Physical Activity	International Physical Activity Questionnaire (IPAQ) reported as average hours and minutes spent participating in moderate to vigorous activity, and the number of participants engaging in at least moderate activity levels (3 days or more of vigorous activity for >20 minutes/day OR 5 days of moderate-intensity activity/walking >30 minutes/day OR >5 days of any activity combination accumulating at least 600 MET-min/week).	0, 6, 12, 26, 52
Body Mass Index	Calculated using weight (kg) / height (m^2) (height collected at baseline only).	
Nutrition Quality	21 item Food Frequency Questionnaire of intake over the past month. (response options for fruit vegetable, discretionary choices, wholegrains and dairy categories: rarely or never, less than once a week, once a week, 2-3 times a week, 4-6 times a week, 1-2 times a day, 3-4 times a day, 5+ a day and response options for meat categories: rarely or never, less than once a week, once a week, 2-3 times a week, 4-6 times a week, 7+ times a week).	0, 6, 12, 26, 52
Sleep Quality	Item-6 from the Pittsburgh Sleep Quality Index (response options: very bad, fairly bad, fairly good, very good).	0, 6, 12, 26, 52
Pain Self Efficacy	2 item Pain Self-Efficacy Questionnaire (PSEQ-2) on a scale of 0-6 with zero indication not at all confident and 6 completely confident.	0, 6, 12, 26, 52
Psychological Distress	Kessler 6 Psychological Distress Scale, as how often a feeling was experiences over the past 30 days (response options: all of the time, most of the time, some of the time, a little of the time, none of the time.	0, 6, 12, 26, 52
Alcohol Consumption	2 questions from the Alcohol Use Disorders Identification Test (AUDIT-C).	0, 6, 12, 26, 52
**Economic outcomes**		
Health care, medication and carer use	Self-reported inventory of health care, community/carer/homecare services and medication use for back pain over the last 6 weeks.	0, 6, 12, 26, 52
Surgical services use	Four yes/no questions assessing participant reported appropriateness of, referral, consultation for, or receipt of surgical procedure.	52
Work absenteeism and presenteeism	Self-reported number of days off work due to back pain (absenteeism), and days at work despite feeling ill, (presenteeism) over the last 6 weeks.	0, 6, 12, 26, 52

© 2025 Mudd E et al. *JAMA Network Open.*

**Safety outcomes**		
Adverse events	Participant reported experience of new medical conditions or an exacerbation of an existing condition (yes/no, with open text).	6, 12, 26, 52
**Process outcomes**		
Participant satisfaction	Participant reported overall satisfaction with the program using a 0-6 Likert scale (0 not at all satisfied, 6 extremely satisfied).	12, 26
Participant intervention fidelity	Participant attendance at clinic appointments, use of resources and telephone services (service provided metrics).	Over 26 weeks
Clinician intervention fidelity	Clinician reported delivery of intervention components per participants using an intervention checklist.	0, 3, 6, 12 weeks

© 2025 Mudd E et al. *JAMA Network Open.*

eTable 2. Participant Fidelity (Adherence and Participation), Reasons for Withdrawal and Satisfaction With Care

**Compliance component**	**HeLP (N=172)**	**Guideline Care (N=172)**
Consultations Attended (y)		
- Initial consultation	147/172 (85%)	133/172 (77%)
- Week 3 physiotherapy	116/172 (67%)	86/172 (50%)[a]
- Week 3 dietitian	121/172 (70%)	-
- Week 6 physiotherapy	89/172 (52%)	-
- Week 12 physiotherapy	71/172 (41%)	48/172 (28%)[b]
Mean consultations attended	3.2 (1.9); n=172	1.8 (1.4); n=172
Viewed chronic pain video (y)	79/172 (46%)	NA
Logged onto online portal	10/172 (6%)	NA
Referred to GHS (y)	147/172 (85%)	NA
Enrolled/completed at least one call with GHS	82/147 (56%)	NA
Median number of calls completed with GHS (accepted at least one call)	3 (IQR 1 to 8); n=82	NA
Graduated GHS (y)	40/147 (27%)	NA
Referrals to Quitline, smokers only (y)	10/46 (21%)	NA
Number of completed Quitline calls[c] (y)		
- Zero	4/7 (57%)	NA
- Two	2/7 (29%)	NA
- 5 or more	1/7 (14%)	NA
Intervention withdrawal reasons		
- Dissatisfaction	8/39 (21%)	8/34 (24%)
- Refused	10/39 (26%)	9/34 (26%)
- Deceased	1/39 (3%)	1/34 (3%)
- Getting other care	3/39 (8%)	2/34 (6%)
- Moved interstate	2/39 (5%)	NA
- Other illnesses	3/39 (8%)	5/34 (15%)
- Other commitments / time	12/39 (31%)	7/34 (21%)
- Other	NA	2/34 (6%)
Satisfaction score, 0-6		
Week 12	4.6 (1.4); n=113	4.0 (2.0); n=126
Week 26	4.3 (1.7); n=123	4.1 (1.9); n=131

Data extracted from intervention logs and service records systematically completed by clinicians and health coaches, and web metrics. Data are n/N (%), mean (SD); or median (IQR); [a]number having at least 2 appointments, [b]number having at least 3 appointments by 12 weeks; [c]3 participants referred to Quitline did not answer. GHS; Get Healthy Service, SD; standard deviation, y; yes.

© 2025 Mudd E et al. *JAMA Network Open.*

eTable 3. Clinician Reported Intervention Fidelity

**Outcome**	**Offered intervention (N=172)**
**Initial (n=147 completed) 85.4%**	
**1.** Completed physical assessment	138/147 (94%)
- Height measured	138/147 (94%)
- Weight measured	139/147 (95%)
**2.** Clinician explained HeLP program components to participant	
- Number of consultations	142/147 (97%)
- Data expectations	146/147 (99%)
- Physio and dietitian contact	146/147 (99%)
- Supplemental telephone services	142/147 (97%)
**3.** Provided low back pain/neuroscience education	147/147 (100%)
**4.** Linked pain to lifestyle behaviours	144/147 (98%)
**5.** Discuss GHS role and offer referral	144/147 (98%)
**6.** Described QL role (smokers only)	32/35 (91%)
**7.** Reassurance of GP involvement	143/147 (97%)
**8.** Established stage and motivations for change	145/147 (99%)
**9.** Acknowledged barriers to change	144/147 (98%)
**10.** Goal setting	144/147 (98%)
Appointment length, minutes, Mean (SD)	66.4 (12.6)
**Total components delivered, Mean %**	**97%**
**Week 3 Physio (n=116 completed)**	
**1.** Recap HeLP program content, education and information from previous session	112/116 (97%)
**2.** Update on GHS and encourage and support participation	107/116 (92%)
**3.** Review goals and homework	115/116 (99%)
**4.** Acknowledge and address barriers to lifestyle change,	113/116 (98%)
**5.** Discuss smoking cessation (smokers only)	23/29 (79%)
**6.** Goal setting	111/116 (96%)
Appointment length (mins), Mean (SD)	34.0 (10.6)
**Total components delivered out of 6 Mean %**	**95%**
**Week 3 Dietitian (n=121 completed)**	
**1.** Recap HeLP program and importance of diet in lifestyle change for back pain management and chronic disease prevention	117/121 (97%)
**2.** Healthy eating education	119/121 (98%)
**3.** Address stage of change and motivations for diet management	114/121 (94%)
**4.** Use patient baseline data or brief assessment to help guide conversation and negotiate changes in current diet patient	114/121 (94%)
**5.** Negotiate strategies to improve diet	115/121 (95%)
**6.** Acknowledge and address barriers to lifestyle change	102/121 (84%)
**7.** Update on the Get Healthy Service and encourage and support participation	111/121 (92%)
**8.** Goal setting	102/121 (84%)
Appointment length (mins), Mean (SD)	38.2 (11.5)
**Total components delivered out of 8 Mean %**	**92%**
**Week 6 Physiotherapy (n=89 completed)**	
**1.** Recap on HELP program content, education and information from previous session	88/89 (99%)
**2.** Update on the Get Healthy Service and encourage and support participation	88/89 (99%)
**3.** Review goals and homework	88/89 (99%)
**4.** Acknowledge and address barriers to lifestyle change	89/89 (100%)
**5.** Discuss smoking cessation (smokers only)	17/21 (81%)
**6.** Goal Setting	84/89 (94%)
Appointment length (mins) Mean (SD)	34.5 (11%)
**Total components delivered out of 6 Mean %**	**97%**

© 2025 Mudd E et al. *JAMA Network Open.*

Clinician reported intervention fidelity continued…	
**Outcome**	**Offered intervention (N=172)**
**Week 12 physiotherapy (n=71 completed)**	
**1.** Reinforce HeLP principles and ask about patient progress/ experience<br>**2.** Completed physical assessment, weight	66/71 (93%)<br>65/71 (92%)
**3.** Update on the Get Healthy Service; encourage and support participation	67/71 (94%)
**4.** Review goals and homework	68/71 (96%)
**5.** Acknowledge and address barriers to lifestyle change	67/71 (94%)
**6.** Smoking cessation referral (smokers only)	5/18 (28%)
**7.** Goal Setting for continued progress/maintenance and long term self-management	66/71 (93%)
Appointment length (mins)	33.2 (10.6)
**Total components delivered out of 6 Mean %**	**92%**

Data captured by an intervention checklist completed by clinicians follow each consultation.
Data are mean (SD), n/N (%).

© 2025 Mudd E et al. *JAMA Network Open.*

eTable 4. Moderation Analyses - Difference in Mean RMDQ Score Between Groups by BMI Category

	HeLP (N=172)	Guideline care (N=172)	Mean Difference (95%CI)	P value
**BMI overall** Three-way interaction p=0.203				
**BMI >25kg/m2**				
Baseline	14.8 (5.3); n=155	14.1 (5.4); n=157	NA	NA
Week 6	12.7 (5.9); n=120	13.2 (6.0); n=142	-1.5 (-2.4 to-0.6)	0.002
Week 12	12.2 (6.2); n=114	12.8 (6.5); n=130	-1.6 (-2.7 to-0.4)	0.007
Week 26	11.7 (6.5); n=128	12.4 (6.8); n=135	-1.1 (-2.3 to 0.1)	0.066
Week 52	11.5 (6.9); n 120	11.5 (7.2); n=131	-0.6 (-1.9 to 0.7)	0.35
**BMI 18.5-<25kg/m2**				
Baseline	13.5 (5.8); n=17	13.5 (6.1); n=15	NA	NA
Week 6	11.2 (6.8); n=13	14.7 (5.8); n=12	-2.25 (-5.2 to 0.7)	0.13
Week 12	10.6 (6.4); n=15	12.5 (5.1); n=12	-1.48 (-4.9 to 1.9)	0.39
Week 26	8.9 (6.5); n=14	13.2 (5.7); n=12	-3.04 (-6.8 to 0.7)	0.12
Week 52	8.7 (7.9); n=14	14.2 (6.6); n=12	-4.63 (-8.5 to -0.7)	0.021

Data are mean (SD); number of participants or mean difference (95% Confidence Interval).

© 2025 Mudd E et al. *JAMA Network Open.*

eTable 5. Characteristics of Compliers and Noncompliers for the HeLP Intervention

	**Compliers[a] N=47**	**Non compliers N=125**	**P value**
**Age, years**	53 (14)	48 (13)	0.046
**Sex, female**	26 (55%)	76 (61%)	0.51
**Employment status**			
Employed or self-employed	19 (40%)	62 (50%)	0.41
**Income**			
Negative or nil	1 (2%)	2 (2%)	0.94
Up to $33,799 per year	19 (40%)	42 (34%)	
Between $33,800-$88,399 per year	19 (40%)	56 (45%)	
Between $88,400 - $207,999 per year	5 (11%)	17 (14%)	
≥$208,000 per year	NA	1 (1%)	
Don’t know	3 (6%)	7 (6%)	
**Private Health Insurance, yes**	16 (34%)	29 (23%)	0.15
**Back pain duration, years**	11.7 (12.7)	10.4 (9.8)	0.46
**Episodes of back pain which have recovered**	3.0 (7.6)	3.2 (13.4)	0.95
**Back pain compensable, yes**	2 (4%)	8 (6.0%)	0.59
**Back pain with leg involvement, yes**	37 (79%)	94 (75%)	0.63
**Have co-existing medical condition, yes**	34 (72%)	96 (77%)	0.54
**Disability RMDQ score, 0-24,**	15.4 (5.8)	14.4 (5.2)	0.28
**Pain scale score, 0-10**	6.7 (1.9)	6.5 (1.6)	0.65
**Self-reported weight, kg**	93.5 (22.5)	89.1 (21.7)	0.24
**Objective measured weight, kg**	96.1 (23.8)	90.6 (23.7)	0.20
**BMI, kg/m^2**	32.1 (8.5)	31.2 (7.4)	0.47
**Quality of life**			
Physical component score	48.4 (8.6)	48.9 (9.6)	0.75
Mental component score	51.7 (9.4)	49.3 (10.5)	0.16
**Smoker, yes**	10 (21%)	36 (29%)	0.32
**Physical activity levels**			
MET minutes/ week	937.6 (1209.0)	985.2 (1483.5)	0.85
Low activity levels	24 (52%)	75 (60%)	
Moderate activity levels	17 (37%)	33 (26%)	
High activity levels	5 (11%)	17 (14%)	
**Nutrition Diet quality score, 5-15**	10.5 (1.6)	10.5 (1.6)	0.91
**Poor sleep quality, yes**	34 (72%)	87 (70%)	0.73
**Pain self-efficacy score, 0-12**	7.4 (3.2)	7.5 (3.2)	0.96
**Psychological distress score, 0-24**	13.6 (5.8)	14.7 (6.2)	0.29
**Risky alcohol consumption**	13 (28%)	30 (24%)	0.62
**Using medication for back pain**	38 (81%)	112 (90%)	0.13
**Sought other care for back pain**	28 (60%)	73 (58%)	0.89
**Using community or carer support for back pain**	19 (49%)	44 (41%)	0.39
**Days off work due to back pain (last 6 weeks)**	0.0 (IQR 0.0 to 1.0)	0.00 (IQR 0.0 to 2.0)	0.51
**Days at work ill due to back pain (last 6 weeks)**	7.0 (IQR 3.0 to 24.0)	6.0 (IQR 3.0 to 17.0)	0.66

Data are mean (SD) or n (%), or median (Interquartile range).[a] Compliance defined as attendance to at least two consultations and completion of 5 or more calls or graduation from the Get Healthy Service. RMDQ; Roland Morris Disability Questionnaire. BMI; Body Mass Index. MET; Metabolic Equivalent of Task.

© 2025 Mudd E et al. *JAMA Network Open.*

eTable 6. Sensitivity Analysis - Difference in the Mean Imputed RMDQ Score Between Treatment Groups

Timepoint	HeLP (N=172)	Guideline care (N=172)	Mean Difference intervention – control (95%CI)	P value
Baseline	14.7 (5.3)	14.0 (5.5)	NA	NA
Week 6	12.4 (5.9)	13.4 (5.9)	-1.5 (-2.4 to -0.6)	0.001
Week 12	12.0 (6.2)	13.0 (6.3)	-1.52 (-2.52 to -0.52)	0.003
Week 26	11.7 (6.5)	12.5 (6.7)	-1.32 (-2.33 to -0.24)	0.016
Week 52	11.2 (6.9)	11.7 (7.1)	-1.00 (-2.21 to 0.21)	0.106

Data are the mean (SD).

© 2025 Mudd E et al. *JAMA Network Open.*

eTable 7. Sensitivity Analysis - Difference in the Mean RMDQ Score Between Treatment Groups Adjusted for Imbalanced Prognostic Variables

Timepoint	HeLP (N=172)	Guideline care (N=172)	Mean Difference intervention – control (95%CI)	P value
Baseline	14.7 (5.4)	14.09 (5.5)	NA	NA
Week 6	12.5 (6.0)	13.3 (6.0)	-1.56 (-2.46 to -0.66)	<0.001
Week 12	12.0 (6.2)	12.8 (6.4)	-1.54 (-2.61 to -0.46)	0.005
Week 26	11.4 (6.5)	12.5 (6.7)	-1.31 (-2.47 to -0.16)	0.026
Week 52	11.2 (7.0)	11.8 (7.2)	-0.91 (-2.12 to 0.30)	0.14

Data are the mean (SD), model if adjusted for baseline RMDQ, whether backpain was compensable, when backpain started and BMI bracket.

© 2025 Mudd E et al. *JAMA Network Open.*

eTable 8. Common Medications Classifications Used for Back Pain by Group

**Medication Category**	**HeLP (N=172)**	**Guideline Care (N=172)**
**Baseline, used medication**	**N=150**	**N=146**
Analgesic (paracetamol)	78/150 (52%)	69/146 (47%)
NSAID	58/150 (39%)	55/146 (38%)
Opioid	42/150 (28%)	43/146 (29%)
Other analgesic	2/150 (1%)	NA
Analgesic (paracetamol with NSAID)	2/150 (1%)	2/146 (1%)
Analgesic (paracetamol with opioid)	32/150 (21%)	31/146 (21%)
Analgesic (combination other than paracetamol) with opioid	NA	2/146 (1%)
Antidepressant	6/150 (4%)	9/146 (6%)
Antiepileptic	31/150 (21%)	20/146 (14%)
Antibiotic	1/150 (1%)	NA
Cannaboid	1/150 (1%)	NA
Corticosteroid	NA	2/146 (1%)
Drugs used in addictive disorders	NA	1/146 (1%)
Gout	NA	1/146 (1%)
Muscle relaxant	10/150 (7%)	10/146 (7%)
Other supplement or mineral	1/150 (1%)	2/146 (1%)
Tetracycline	1/150 (1%)	NA
Topical	13/150 (9%)	18/146 (12%)
**Week 6, used medication**	**N=79**	**N=91**
Analgesic (paracetamol)	38/79 (48%)	39/91 (43%)
NSAID	31/79 (39%)	30/91 (33%)
Opioid	22/79 (28%)	35/91 (39%)
Other analgesic	1/79 (1%)	2/91 (2%)
Analgesic (paracetamol with NSAID)	NA	2/91 (2%)
Analgesic (paracetamol with opioid)	20/79 (25%)	23/91 (25%)
Analgesic (combination other than paracetamol) with opioid	2/79 (3%)	1/91 (1%)
Antidepressant	9/79 (7%)	9/91 (10%)
Antiepileptic	22/79 (11%)	19/91 (20%)
Antibiotic	NA	NA
Antipsychotic	3/79 (4%)	NA
Antipropulsive	1/79 (1%)	NA
Cannaboid	2/79 (3%)	NA
Corticosteroid	1/79 (1%)	1/91 (1%)
Dopaminergic	NA	1/91 (1%)
Drugs used in addictive disorders	NA	NA
Gout	NA	NA
Hypnotics and sedative	NA	1/91 (1%)
Muscle relaxant	5/79 (6%)	6/91 (7%)
Other supplement or mineral	4/79 (5%)	2/91 (2%)
Tetracycline	1/79 (1%)	NA
Topical	6/79 (8%)	3/91 (3%)
**Week 12, used medication**	**N=81**	**N=86**
Analgesic (paracetamol)	44/81 (54%)	35/86 (41%)
NSAID	24/81 (30%)	25/86 (29%)
Opioid	29/81 (36%)	29/86 (34%)
Other analgesic	1/81 (1%)	1/86 (1%)
Analgesic (paracetamol with NSAID	NA	2/86 (2%)
Analgesic (paracetamol with opioid)	17/81 (21%)	18/86 (21%)
Analgesic (combination other than paracetamol) with opioid	1/81 (1%)	2/86 (2%)
Antidepressant	5/81 (6%)	9/86 (10%)
Antiepileptic	18/81 (22%)	15/86 (17%)

© 2025 Mudd E et al. *JAMA Network Open.*

**Medication Category continued…**	**HeLP (N=172)**	**Guideline Care (N=172)**
**Week 12, used medication**	**N=81**	**N=86**
Antibiotic	NA	NA
Cannaboid	NA	NA
Corticosteroid	NA	1/86 (1%)
Drugs used in addictive disorders	NA	NA
Gout	NA	1/86 (1%)
Muscle relaxant	3/81 (4%)	4/86 (5%)
Other supplement or mineral	2/81 (3%)	NA
Tetracycline	NA	NA
Topical	4/81 (5%)	7/86 (8%)
**Week 26, used medication**	**N=83**	**N=84**
Analgesic (paracetamol)	37/83 (45%)	37/84 (44%)
NSAID	38/83 (46%)	22/84 (26%)
Opioid	27/83 (33%)	30/84 (36%)
Other analgesic	2/83 (2%)	1/84 (1%)
Analgesic (paracetamol with NSAID)	NA	3/84 (4%)
Analgesic (paracetamol with opioid)	16/83 (19%)	19/84 (23%)
Analgesic (combination other than paracetamol) with opioid	1/83 (1%)	3/84 (7%)
Antidepressant	6/83 (7%)	10/84 (12%)
Antiepileptic	20/83 (24%)	14/84 (17%)
Antibiotic	NA	NA
Antiobesity	1/83 (1%)	NA
Cannaboid	NA	NA
Corticosteroid	NA	1/84 (1%)
Drugs used in addictive disorders	NA	NA
Gout	NA	NA
Hypnotics and sedative	1/83 (1%)	1/84 (1%)
Muscle relaxant	3/83 (4%)	7/84 (8%)
Other supplement or mineral	2/83 (2%)	1/84 (1%)
Tetracycline	NA	NA
Topical	3/83 (4%)	6/84 (7%)
**Week 52, used medication**	**N=72**	**N=85**
Analgesic (paracetamol)	34/72 (47%)	37/85 (43%)
NSAID	22/72 (31%)	25/85 (29%)
Opioid	28/72 (39%)	27/85 (32%)
Other analgesic	1/72 (1%)	1/85 (1%)
Analgesic (paracetamol with NSAID)	NA	3/85 (4%)
Analgesic (paracetamol with opioid)	15/72 (20%)	14/85 (16%)
Analgesic (combination other than paracetamol) with opioid	3/72 (4%)	NA
Antidepressant	13/72 (18%)	14/85 (16%)
Antiepileptic	17/72 (24%)	14/85 (16%)
Anxiolytics	1/72 (1%)	NA
Antibiotic	NA	NA
Cannaboid	NA	NA
Corticosteroid	NA	1/85 (1%)
Drugs used in addictive disorders	NA	NA
Gout	NA	NA
Hypnotics and sedative	1/72 (1%)	1/85 (1%)
Muscle relaxant	1/72 (1%)	4/85 (5%)
Other supplement or mineral	5/72 (7%)	NA
Tetracycli1ne	1/72 (1%)	NA
Topical	7/72 (10%)	4/85 (5%)

Data are the n/N (%) of participants who reported they were currently taking medications for their back pain. NSAID; non-steroidal anti-inflammatory drug. Topical; any ointment or cream applied to skin including NSAID agent.

© 2025 Mudd E et al. *JAMA Network Open.*

eTable 9. Common Healthcare Services Used for Low Back Pain

**Service Category**	**HeLP (N=172)**	**Guideline Care (N=172)**
**Baseline, used other healthcare**	**N=101**	**N=95**
Alternative medicine	1/101 (1%)	5/95 (5%)
Chiropractor	7/101(7%)	7/95 (7%)
Dietitian	1/101 (1%)	NA
ED presentation	2/101 (2%)	NA
General practitioner	62/101 (61%)	66/95 (69%)
Hospital admission	1/101 (1%)	1/95 (1%)
Imaging	4/101 (4%)	4/95 (4%)
Massage therapy	13/101 (13%)	10/95 (11%)
Neurosurgical specialist	2/101 (2%)	5/95 (5%)
Orthopaedic specialists	2/101 (2%)	NA
Pain clinic	3/101 (3%)	2/95 (2%)
Physical activity services	3/101 (3%)	NA
Physiotherapy	20/101 (12%)	10/95 (11%)
Spinal injection	3/101 (3%)	2/95 (2%)
Other allied health	4/101 (4%)	2/95 (2%)
Other medical specialist	4/101 (4%)	5/95 (5%)
**Week 6, used other healthcare**	**N=65**	**N=66**
Alternative medicine	3/65 (5%)	2/66 (3%)
Chiropractor	4/65 (6%)	7/66 (10%)
Dietitian	10/65 (15%)	1/66 (1%)
ED presentation	1/65 (1%)	1/66 (1%)
General Practitioner	34/65 (52%)	37/66 (56%)
Hospital admission	1/65 (1%)	2/66 (3%)
Imaging	3/65 (5%)	2/66 (3%)
Massage therapy	8/65 (12%)	6/66 (9%)
Neurosurgical specialist	1/65 (1%)	1/66 (1%)
Orthopaedic specialists	NA	1/66 (1%)
Pain clinic	3/65 (5%)	3/66 (5%)
Physical activity services	4/65 (6%)	2/66 (3%)
Physiotherapy	16/65 (25%)	16/66 (24%)
Spinal injection	3/65 (5%)	1/66 (1%)
Surgery	1/65 (1%)	NA
Other allied health	NA	4/66 (6%)
Other medical specialist	4/65 (6%)	5/66 (8%)
Other	2/65 (3%)	2/66 (3%)
**Week 12, used other healthcare**	**N=58**	**N=43**
Alternative medicine	3/58 (5%)	NA
Chiropractor	2/58 (3%)	3/43 (7%)
Community service	1/58 (2%)	NA
Dietitian	9/58 (16%)	1/43 (2%)
ED presentation	1/58 (2%)	NA
General Practitioner	33/122 (27%)	29/43 (67%)
Hospital admission	1/58 (2%)	2/43 (5%)
Imaging	NA	3/43 (7%)
Massage therapy	8/ (14%)	3/43 (7%)
Orthopaedic specialists	1/58 (2%)	NA
Pain clinic	3/58 (5%)	1/43 (2%)
Physical activity services	3/58 (5%)	1/43 (2%)
Physiotherapy	10/58 (17%)	7/43 (16%)
Spinal injection	1/58 (2%)	2/43 (5%)
Surgery	NA	1/43 (2%)
Other allied health	3/58 (5%)	2/43 (5%)

© 2025 Mudd E et al. *JAMA Network Open.*

**Service Category continued…**	**HeLP**	**Guideline Care**
**Week 12, used other healthcare**	**N=58**	**N=43**
Other medical specialist	4/58 (7%)	5/43 (12%)
Other	1/58 (2%)	3/43 (7%)
**Week 26, used other healthcare**	**N=59**	**N=35**
Alternative medicine	1/59 (2%)	1/35 (3%)
Chiropractor	3/59 (5%)	4/131 (11%)
Community service	1/59 (2%)	NA
Dietitian	2/59 (3%)	NA
ED presentation	1/59 (2%)	1/35 (3%)
General Practitioner	33/59 (56%)	27/35 (77%)
Hospital admission	1/59 (2%)	NA
Imaging	2/59 (3%)	NA
Massage therapy	13/59 (22%)	2/35 (6%)
Orthopaedic specialists	4/59 (7%)	NA
Neurosurgical specialist	1/59 (2%)	NA
Pain clinic	3/59 (5%)	1/35 (3%)
Physical activity services	3/59 (5%)	2/35 (6%)
Physiotherapy	10/59 (17%)	5/35 (14%)
Spinal injection	2/59 (3%)	2/35 (6%)
Surgery	1/59 (2%)	NA
Other allied health	4/59 (7%)	3/35 (9%)
Other medical specialist	3/59 (5%)	2/35 (6%)
Other	1/59 (2%)	NA
**Week 52, used other healthcare**	**N=48**	**N=44**
Alternative medicine	1/48 (2%)	1/44 (2%)
Chiropractor	3/48 (6%)	9/44 (20%)
ED presentation	1/48 (2%)	NA
General Practitioner	24/48 (50%)	23/44 (52%)
Imaging	2/48 (4%)	NA
Massage therapy	9/48 (19%)	7/44 (16%)
Orthopaedic specialists	2/48 (4%)	1/44 (2%)
Neurosurgical specialist	1/48 (2%)	NA
Pain clinic	NA	2/44 (3%)
Physical activity services	2/48 (4%)	2/44 (3%)
Physiotherapy	6/48 (12%)	4/44 (9%)
Spinal injection	1/48 (2%)	3/44 (7%)
Surgery	NA	1/44 (2%)
Other allied health	1/48 (2%)	3/44 (7%)
Other medical specialist	6/48 (13%)	NA
Other	1/48 (2%)	1/44 (2%)
Referred to surgeon (y)	8/130 (6%)	8/130 (6%)
Attended appointment with surgeon (y)	20/130 (15%)	10/130 (8%)
Had back surgery (y)	15/130 (12%)	16/132 (12%)
Believe surgery is appropriate (y)	26/127 (20%)	34/127 (27%)

Data are the n/N (%) of participants who reported they used health care services for their back pain in the last 6 weeks.

© 2025 Mudd E et al. *JAMA Network Open.*

eTable 10. Carer or Community Services Support Used

Service Category	HeLP (N=172)	Guideline care (N=172)
**Baseline, used service**	**N=63**	**N=52**
Family member	53/63 (84%)	36/52 (69%)
Friend/ neighbour	7/63 (11%)	7/52 (14%)
Cleaner Services	5/63 (8%)	8/52 (15%)
Lawn mowing/ Gardening services	1/63 (2%)	1/52 (2%)
Driving services (e.g. taxi)	NA	NA
Nursing	NA	1/52 (2%)
Meal provision services (e.g. Meals on Wheels)	NA	NA
Other (e.g. healthcare workers, employees)	5/63 (8%)	2/52 (4%)
**Week 6, used service**	**N=44**	**N=44**
Family member	36/44 (81%)	29/44 (66%)
Friend/ neighbour	5/44 (11%)	5/44 (18%)
Cleaner Services	5/44 (11%)	8/44 (18%)
Lawn mowing/ Gardening services	2/44 (4%)	2/44 (5%)
Driving services (e.g. taxi)	NA	NA
Nursing	1/44 (2%)	NA
Meal provision services (e.g. Meals on Wheels)	NA	NA
Other (e.g. healthcare workers, employees)	3/44 (7%)	4/44 (9%)
**Week 12, used service**	**N=34**	**N=31**
Family member	23/34 (68%)	20/31 (64%)
Friend/ neighbour	7/34 (21%)	7/31 (23%)
Cleaner Services	4/34 (12%)	5/31 (16%)
Lawn mowing/ Gardening services	1/34 (3%)	2/31 (7%)
Driving services (e.g. taxi)	1/34 (3%)	1/31 (3%)
Nursing	2/34 (6%)	NA
Meal provision services (e.g. Meals on Wheels)	1/34 (3%)	NA
Other (e.g. healthcare workers, employees)	4/34 (9%)	1/31 (3%)
**Week 26, used service**	**N=37**	**N=25**
Family member	29/37 (78%)	20/25 (80%)
Friend/ neighbour	3/37 (8%)	3/25 (12%)
Cleaner Services	4/37 (11%)	2/25 (8%)
Lawn mowing/ Gardening services	1/37 (3%)	NA
Driving services (e.g. taxi)	NA	NA
Nursing	1/37 (3%)	NA
Meal provision services (e.g. Meals on Wheels)	NA	NA
Other (e.g. healthcare workers, employees)	1/37 (3%)	2/25 (8%)
**Week 52, used service**	**N=41**	**N=32**
Family member	28/41 (68%)	19/32 (59%)
Friend/ neighbour	6/41 (15%)	9/32 (28%)
Cleaner Services	4/41 (10%)	4/32 (13%)
Lawn mowing/ Gardening services	4/41 (10%)	1/32 (3%)
Driving services (e.g. taxi)	1/41 (2%)	NA
Nursing	1/41 (2%)	1/32 (3%)
Meal provision services (e.g. Meals on Wheels)	NA	NA
Other (e.g. healthcare workers, employees)	3/41 (7%)	2/32 (6%)

Data are the n/N (%) of participants who reported they had someone help them with tasks at home they couldn’t do because of their back pain in the last 6 weeks.

© 2025 Mudd E et al. *JAMA Network Open.*

eTable 11. Activities Requiring Help With From Carers or Community Support

Task Category	HeLP (N=172)	Guideline care (N=172)
**Baseline, help required**	**N=63**	**N=52**
Childcare	1/63 (2%)	3/52 6%)
Cleaning	16/63 (25%)	12/52 (23%)
Daily care	8/63 (13%)	4/52 (8%)
Gardening	16/63 (25%)	11/52 (21%)
General	41/63 (65%)	27/52 (52%)
Groceries	3/63 (5%)	3/52 (6%)
Home reno	1/63 (2%)	NA
Lifting	4/63 (6%)	5/52 (10%)
Movement	NA	1/52 (2%)
Transport	9/63 (14%)	4/52 (8%)
Other	NA	NA
**Week 6, help required**	**N=44**	**N=44**
Childcare	NA	NA
Cleaning	15/44 (34%)	20/44 (46%)
Daily care	5/44 (11%)	3/44 (7%)
Gardening	11/44 (25%)	10/44 (23%)
General	20/44 (46%)	17/44 (39%)
Groceries	7/44 (16%)	6/44 (14%)
Home reno	NA	2/44 (5%)
Lifting	3/44 (7%)	3/44 (7%)
Movement	NA	1/44 (2%)
Transport	6/44 (14%)	6/44 (14%)
Other	2/44 (5%)	NA
**Week 12, help required**	**N=34**	**N=31**
Childcare	NA	NA
Cleaning	12/34 (35%)	15/31 (48.4)
Daily care	2/34 (6%)	2/31 (6.5)
Gardening	8/34 (24%)	7/31 (22.6)
General	17/34 (50%)	10/31 (32.3)
Groceries	7/34 (21%)	3/31 (9.1)
Home reno	NA	1/31 (3.2)
Lifting	4/34 (12%)	2/31 (6.5)
Movement	NA	NA
Transport	7/34 (21%)	4/31 (12.9)
Other	1/34 (3%)	NA
**Week 26, help required**	**N=37**	**N=25**
Childcare	NA	1/25 (4%)
Cleaning	15/37 (41%)	9/25 (36%)
Daily care	2/37 (5%)	1/25 (4%)
Gardening	8/37 (22%)	5/25 (20%)
General	16/37 (43%)	13/25 (52%)
Groceries	7/37 (19%)	8/25 (32%)
Home reno	NA	NA
Lifting	4/37 (11%)	NA
Movement	NA	NA
Transport	3/37 (8%)	4/25 (16%)
Other	1/37 (3%)	NA
**Week 52, help required**	**N=41**	**N=32**
Childcare	NA	NA
Cleaning	16/41 (39%)	14/32 (44%)
Daily care	3/41 (7%)	3/32 (9%)

© 2025 Mudd E et al. *JAMA Network Open.*

**Task Category continued…**	**HeLP**	**Guideline care**
**Week 52, help required**	**N=41**	**N=32**
Gardening	11/41 (27%)	10/32 (31%)
General	13/41 (32%)	6/32 (19%)
Groceries	5/41 (12%)	7/32 (22%)
Home reno	NA	1/32 (3%)
Lifting	6/41 (15%)	4/32 (13%)
Movement	NA	NA
Transport	7/41 (17%)	5/32 (16%)
Other	2/41 (5%)	NA

Data are the n/N (%) of participants who reported they had someone help them with tasks at home they couldn't do because of their back pain in the last 6 weeks.

© 2025 Mudd E et al. *JAMA Network Open.*

eTable 12. Frequency of Adverse Events (*IDC10* Categories) by Group

**ICD codes**	**HeLP (N=172)**	**Guideline care (N=172)**
**Week 6, condition reported**	**N=22**	**N=22**
1B70 Bacterial cellulitis, erysipelas or lymphangitis	1/22 (5%)	NA
1E32 Influenza, virus not identified	NA	1/22 (5%)
1E91 Zoster	1/22 (5%)	NA
2F9Z neoplasms of unknown behaviour of unspecified site	NA	1/22 (5%)
3B64 Thrombocytopenia	1/22 (5%)	NA
5A02 Thyrotoxicosis	NA	1/22 (5%)
6A6Z Bipolar or related disorders, unspecified	1/22 (5%)	NA
8B93 Radiculopathy	NA	1/22 (5%)
8C0Z Polyneuropathy, unspecified	NA	1/22 (5%)
BC4Z Diseases of the myocardium or cardiac chambers, unspecified	1/22 (5%)	NA
BC81 Supraventricular tachyarrhythmia	NA	1/22 (5%)
BD54 Diabetic foot ulcer	NA	1/22 (5%)
CA0A Chronic rhinosinusitis	1/22 (5%)	NA
CA40 Pneumonia	1/22 (5%)	NA
FA01 Osteoarthritis of knee	NA	1/22 (5%)
FA25 Gout	NA	1/22 (5%)
FA36 Effusion of joint	NA	1/22 (5%)
FB56 Specified soft tissue disorders, not elsewhere classified	1/22 (5%)	3/22 (14%)
MB47 Abnormality of tonus and reflex	NA	1/22 (5%)
MD81 Abdominal or pelvic pain	1/22 (5%)	NA
ME82 Pain in joint	3/22 (14%)	NA
ME84 spinal pain	5/22 (23%)	5/22 (23%)
NA80 Superficial injury of thorax	1/22 (5%)	NA
NB52 Fracture of lumbar spine or pelvis	1/22 (5%)	NA
NB9Z Injuries to the abdomen, lower back, lumbar spine or pelvis, unspecified	NA	1/22 (5%)
ND50 Fracture of spine, level unspecified	NA	1/22 (5%)
nE81 Injury or harm arising from a procedure, not elsewhere classified	1/22 (5%)	NA
FB50 Bursitis	NA	1/22 (5%)
8A80 Migraine	1/22 (5%)	NA
5A21 Hypoglycaemia in the context of diabetes mellitus	1/22 (5%)	NA
**Week 12, condition reported**	**N=18**	**N=21**
1B70 Bacterial cellulitis, erysipelas or lymphangitis	NA	1/21 (5%)
3B64 Thrombocytopenia	1/18 (6%)	NA
5A0Y Other specified disorders of the thyroid gland or thyroid hormones system	1/18 (6%)	NA
5A11 Type 2 diabetes mellitus	1/18 (6%)	NA
6A60 Bipolar type I disorder	1/18 (6%)	NA
6A6Z Bipolar or related disorders, unspecified	NA	1/21 (5%)
8B93 Radiculopathy	1/18 (6%)	NA
9B72 Inflammatory diseases of the retina	1/18 (6%)	NA
AB70 Otalgia or effusion of ear	1/18 (6%)	NA
CA07 Acute upper respiratory infections	1/18 (6%)	NA
CA0A Chronic rhinosinusitis	1/18 (6%)	NA
CA23 Asthma	1/18 (6%)	NA
FA0Z Osteoarthritis, unspecified	1/18 (6%)	1/21 (5%)
FA36 Effusion of joint	1/18 (6%)	1/21 (5%)
FB56 Specified soft tissue disorders, not elsewhere classified	2/18 (11%)	NA
FB82 Chondropathies	NA	1/21 (5%)

© 2025 Mudd E et al. *JAMA Network Open.*

ICD codes continued…	HeLP	Guideline care
**Week 12, condition reported**	**N=18**	**N=21**
GB6Z Kidney failure, unspecified	NA	1/21 (5%)
MD81 Abdominal or pelvic pain	1/18 (6%)	NA
ME05 Change in bowel habit	NA	1/21 (5%)
ME82 Pain in joint	NA	2/21 (10%)
ME84 spinal pain	2/18 (11%)	9/21 (43%)
MF50 Abnormal micturition	NA	1/21 (5%)
NA02 Fracture of skull or facial bones	1/18 (6%)	NA
NC16 Injury of muscle, fascia, tendon or bursa at shoulder or upper arm level	NA	1/21 5%)
NC53 Fracture at wrist or hand level	NA	1/21 (5%)
ND13 Fracture of foot, except ankle	NA	1/21 (5%)
MG3Z Pain, unspecified	1/18 (6%)	NA
FB50 Bursitis	1/18 (6%)	NA
DB92 non-alcoholic fatty liver disease	NA	1/21 (5%)
DB60 Haemorrhoids	NA	1/21 (5%)
**Week 26, condition reported**	**N=31**	**N=24**
1H0Z Infection, unspecified	1/31 (3%)	NA
3A9Z Anaemias or other erythrocyte disorders, unspecified	1/31 (3%)	NA
5A02 Thyrotoxicosis	1/31 (3%)	NA
5A11 Type 2 diabetes mellitus	1/31 (3%)	NA
6A7Z Depressive disorders, unspecified	3/31 (10%)	NA
8B93 Radiculopathy	2/31 (6%)	1/24 (4%)
8E49 Postviral fatigue syndrome	1/31 (3%)	NA
BA00 Essential hypertension	NA	1/24 (4%)
BC9Z Cardiac arrhythmia, unspecified	NA	1/24 (4%)
CA22 Chronic obstructive pulmonary disease	1/31 (3%)	NA
CA23 Asthma	1/31 (3%)	NA
CA40 Pneumonia	1/31 (3%)	NA
DB60 Haemorrhoids	NA	1/24 (4%)
DB9Z Diseases of liver, unspecified	NA	2/24 (8%)
DD5Z Hernias, unspecified	NA	1/24 (4%)
FA01 Osteoarthritis of knee	NA	1/24 (4%)
FA2Z Inflammatory arthropathies, unspecified	NA	1/24 (4%)
FA30 Acquired deformities of fingers or toes	1/31 (3%)	NA
FA36 Effusion of joint	NA	1/24 (4%)
FB50 Bursitis	1/31 (3%)	1/24 (4%)
FB53 Shoulder lesions	1/31 (3%)	NA
FB55 Certain specified enthesopathies	1/31 (3%)	NA
FB56 Specified soft tissue disorders, not elsewhere classified	1/31 (3%)	NA
MD81 Abdominal or pelvic pain	1/31 (3%)	NA
ME82 Pain in joint	3/31 (10%)	3/24 (13%)
ME84 spinal pain	9/31 (29%)	7/24 (29%)
NA23 Dislocation or strain or sprain of joints or ligaments at neck level	1/31 (3%)	NA
NB9Z Injuries to the abdomen, lower back, lumbar spine or pelvis, unspecified	NA	1/24 (4%)
NC1Z Injuries to the shoulder or upper arm, unspecified	1/31 (3%)	NA
NC92 Fracture of lower leg, including ankle	NA	1/24 (4%)
ND13 Fracture of foot, except ankle	NA	1/24 (4%)
PA0Z Unintentional land transport traffic event injuring a user of unspecified land transport	NA	1/24 (4%)
QE01 Stress, not elsewhere classified	1/31 (3%)	NA
6B40 Post traumatic stress disorder	1/31 (3%)	NA
5C80 Hyperlipoproteinaemia	NA	1/24 (4%)
FB82 Chondropathies	NA	1/24 (4%)

© 2025 Mudd E et al. *JAMA Network Open.*

ICD codes continued…	HeLP	Guideline care
**Week 26, condition reported**	**N=31**	**N=24**
7B2Z Sleep-wake disorders, unspecified	NA	1/24 (4%)
GC2Z Diseases of the urinary system, unspecified	1/31 (3%)	NA
**Week 52, condition reported**	**N=26**	**N=23**
1G40 Sepsis without septic shock	1/26 (4%)	NA
2B5K Unspecified malignant soft tissue tumours or sarcomas of bone	1/26 (4%)	NA
2C76 Malignant neoplasms of corpus uteri	1/26 (4%)	NA
5A02 Thyrotoxicosis	NA	1/23 (4%)
5A0Z Disorders of the thyroid gland or thyroid hormones system, unspecified	1/26 (4%)	NA
5C80 Hyperlipoproteinaemia	NA	1/23 (4%)
8B93 Radiculopathy	1/26 (4%)	NA
8C0Z Polyneuropathy, unspecified	NA	1/23 (4%)
BC9Z Cardiac arrhythmia, unspecified	NA	1/23 (4%)
CA0A Chronic rhinosinusitis	NA	1/23 (4%)
CA20 Bronchitis	1/26 (4%)	NA
CA22 Chronic obstructive pulmonary disease	1/26 (4%)	NA
CA2Z Lower respiratory tract disease, unspecified	1/26 (4%)	NA
DB9Z Diseases of liver, unspecified	NA	1/23 (4%)
DC10 Acquired anatomical alterations of gallbladder or bile ducts	NA	1/23 (4%)
DD5Z Hernias, unspecified	1/26 (4%)	NA
FA00 Osteoarthritis of hip	NA	1/23 (4%)
FA02 Osteoarthritis of wrist and hand	NA	1/23 (4%)
FA0Z Osteoarthritis, unspecified	NA	1/23 (4%)
FA84 Spondylolisthesis	1/26 (4%)	NA
FA92 Inflammatory spondyloarthritis	1/26 (4%)	NA
FB40 Tenosynovitis	1/26 (4%)	NA
FB50 Bursitis	1/26 (4%)	1/23 (4%)
FB51 Fibroblastic disorders	1/26 (4%)	NA
FB55 Certain specified enthesopathies	1/26 (4%)	NA
FB56 Specified soft tissue disorders, not elsewhere classified	1/26 (4%)	3/23 (13%)
MB21 Symptoms, signs or clinical findings involving cognition	NA	1/23 (4%)
ME82 Pain in joint	1/26 (4%)	2/23 (9%)
ME84 spinal pain	6/26 (23%)	3/23 (13%)
NC16 Injury of muscle, fascia, tendon or bursa at shoulder or upper arm level	1/26 (4%)	1/23 (4%)
NB9Z Injuries to the abdomen, lower back, lumbar spine or pelvis, unspecified	NA	1/23 (4%)
PA0Z Unintentional land transport traffic event injuring a user of unspecified land transport	NA	1/23 (4%)
PA61/ Fall from or through roof	NA	1/23 (4%)
PA6Z Unintentional fall from unspecified height	1/26 (4%)	NA
PK80 Medical or surgical procedure associated with injury or harm in therapeutic use	1/26 (4%)	NA
FB82 Chondropathies	NA	1/23 (4%)
NB53 Dislocation or strain or sprain of joints or ligaments of lumbar spine or pelvis	1/26 (3%)	NA
BE2Y Other specified diseases of the circulatory system	NA	1/23 (4%)

Data are the n/N (%) of participants who reported they had an adverse event (new medical condition or exacerbation of an existing condition) since their last contact with the study team.

© 2025 Mudd E et al. *JAMA Network Open.*

eTable 13. Other Illnesses Requiring Medication or Health Care

**Illness**	**HeLP (N=172)**	**Guideline Care (N=172)**
**Baseline, other illness reported**	**N=130**	**N=119**
Allergies	1/130 (1%)	NA
Arthritis	NA	NA
Asthma	32/130 (25%)	15/119 (13%)
Blood and lymphatic disorder	NA	NA
Blood clot or thrombosis	3/130 (2%)	8/119 (7%)
Cancer	1/130 (1%)	3/119 (2%)
Communicable or infectious disease	1/130 (1%)	1/119 (1%)
Depression/Anxiety/Stress	68/130 (52%)	64/119 (54%)
Diabetes	1/130 (1%)	NA
Gastrointestinal tract condition	6/130 (5%)	10/119 (8%)
Heart disease	NA	3/119 (3%)
High Blood Cholesterol	47/130 (36%)	37/119 (31%)
High Blood Pressure	54/130 (42%)	62/119 (52%)
Kidney conditions	1/130 (1%)	2/119 (2%)
Liver Condition	1/130 (1%)	NA
Multiple Sclerosis	NA	NA
Neurological disorder	3/130 (2%)	NA
Osteoporosis	11/130 (8%)	11/119 (9%)
Other Mental Health condition	2/130 (1%)	3/119 (3%)
Other musculoskeletal conditions	11/130 (8%)	1/119 (1%)
Overweight/Obesity	18/130 (14%)	17/119 (14%)
Pre Diabetes	1/130 (1%)	1/119 (1%)
Reproductive organ conditions	2/130 (1%)	NA
Respiratory Condition	1/130(1%)	1/119 (1%)
Skin or sensory organ condition	NA	NA
Sleep disorders	4/130 (3%)	4/119 (3%)
Stroke	NA	1/119 (1%)
Thyroid/ Endocrine/Hormone disorders	7/130 (5%)	11/119 (9%)
**Week 6, other illness reported**	**N=95**	**N=101**
Arthritis	NA	1/101 (1%)
Asthma	20/95 (21%)	17/101 (17%)
Blood and lymphatic disorder	1/95 (1%)	NA
Blood clot or thrombosis	NA	1/101 (1%)
Communicable or infectious disease	1/95 (1%)	NA
Depression/Anxiety/Stress	47/95 (49%)	55/101 (54%)
Gastrointestinal tract condition	8/95 (8%)	1/101 (1%)
High Blood Cholesterol	29/95 (31%)	31/101 (31%)
High Blood Pressure	44/95 (46%)	53/101 (52%)
Kidney conditions	1/95 (1%)	NA
Neurological disorder	1/95 (1%)	NA
Osteoporosis	11/95 (12%)	8/101 (8%)
Other	NA	1/101 (1%)
Other Mental Health condition	1/95 (1%)	1/101 (1%)
Other musculoskeletal conditions	8/95 (8%)	4/101 (4%)
Overweight/Obesity	17/95 (18%)	17/101 (17%)
Reproductive organ conditions	2/95 (2%)	NA
Respiratory Condition	1/95 (1%)	1/101 (1%)
Skin or sensory organ condition	5/95 (5%)	1/101 (1%)
Sleep disorders	1/95 (1%)	2/101 (2%)
Thyroid/ Endocrine/Hormone disorders	2/95 (2%)	5/101 (5%)
**Week 12, other illness reported**	**N=90**	**N=99**
Asthma	18/90 (20%)	10/99 (10%)

© 2025 Mudd E et al. *JAMA Network Open.*

**Illness continued…**	**HeLP**	**Guideline Care**
**Week 12, other illness reported**	**N=90**	**N=99**
Blood clot or thrombosis	2/90 (2%)	3/99 (3%)
Cancer	NA	1/99 (1%)
Communicable or infectious disease	2/90 (2%)	NA
Depression/Anxiety/Stress	43/90 (48%)	47/99 (47%)
Gastrointestinal tract condition	4/90 (4%)	5/99 (5%)
Heart disease	NA	2/99 (2%)
High Blood Cholesterol	28/90 (31%)	31/99 (31%)
High Blood Pressure	41/90 (46%)	47/99 (47%)
Kidney conditions	1/90 (1%)	NA
Multiple Sclerosis	1/90 (1%)	NA
Osteoporosis	10/90 (11%)	9/99 (9%)
Other Mental Health condition	NA	2/99 (2%)
Other musculoskeletal conditions	7/90 (8%)	5/99 (5%)
Overweight/Obesity	13/90 (14%)	18/99 (18%)
Pre Diabetes	1/90 (1%)	NA
Reproductive organ conditions	1/90 (1%)	NA
Respiratory Condition	1/90 (1%)	NA
Sleep disorders	NA	1/99 (1%)
Thyroid/ Endocrine/Hormone disorders	3/90 (3%)	5/99 (5%)
**Week 26, other illness reported**	**N=84**	**N=91**
Allergies	1/84 (1%)	NA
Arthritis	1/84 (1%)	2/91 (2%)
Asthma	18/84 (21%)	13/91 (14%)
Blood clot or thrombosis	1/84 (1%)	3/91 (3%)
Cancer	1/84 (1%)	1/91 (1%)
Communicable or infectious disease	NA	3/91 (3%)
Depression/Anxiety/Stress	44/84 (52%)	47/91 (52%)
Gastrointestinal tract condition	4/84 (5%)	3/91 (3%)
Heart disease	NA	1/91 (1%)
High Blood Cholesterol	32/84 (38%)	25/91 (27%)
High Blood Pressure	42/84 (50%)	52/91 (57%)
Kidney conditions	1/84 (1%)	1/91 (1%)
Liver Condition	1/84 (1%)	NA
neurological disorder	2/84 (2%)	1/91 (1%)
Osteoporosis	10/84 (12%)	6/91 (7%)
Other Mental Health condition	1/84 (1%)	1/91 (1%)
Other musculoskeletal conditions	7/84 (8%)	3/91 (3%)
Overweight/Obesity	8/84 (10%)	13/91 (14%)
Pre Diabetes	1/84 (1%)	NA
Respiratory Condition	NA	1/91 (1%)
Skin or sensory organ condition	NA	1/91 (1%)
Sleep disorders	1/84 (1%)	1/91 (1%)
Stroke	1/84 (1%)	NA
Thyroid/ Endocrine/Hormone disorders	1/84 (1%)	1/91 (1%)
**Week 52, other illness reported**	**N=89**	**N=98**
Arthritis	NA	2/98 (2%)
Asthma	22/89 (25%)	10/98 (10%)
Blood and lymphatic disorder	1/89 (1%)	1/98 (1%)
Blood clot or thrombosis	3/89 (3%)	2/98 (2%)
Cancer	3/89 (3%)	1/98 (1%)
Communicable or infectious disease	1/89 (1%)	1/98 (1%)
Depression/Anxiety/Stress	50/89 (56%)	53/98 (54%)
Gastrointestinal tract condition	6/89 (7%)	4/98 (4%)
Heart disease	NA	1/98 (1%)
High Blood Cholesterol	26/89 (29%)	32/98 (33%)
High Blood Pressure	38/89 (43%)	54/98 (55%)

© 2025 Mudd E et al. *JAMA Network Open.*

**Illness continued…**	**HeLP**	**Guideline Care**
**Week 52, other illness reported**	**N=89**	**N=98**
Kidney conditions	1/89 (1%)	NA
Osteoporosis	6/89 (7%)	9/98 (9%)
Other Mental Health condition	1/89 (1%)	1/98 (1%)
Other musculoskeletal conditions	2/89 (2%)	4/98 (4%)
Overweight/Obesity	11/89 (12%)	22/98 (22%)
Skin or sensory organ condition	NA	1/98 (1%)
Sleep disorders	2/89 (2%)	NA
Thyroid/ Endocrine/Hormone disorders	2/89 (2%)	6/98 (6%)

Data are the n/N (%) of participants who reported they required medical attention or took medication for another illness in the last two months.

© 2025 Mudd E et al. *JAMA Network Open.*
