# Supplementary material for: Healthy Lifestyle Care vs Guideline-Based Care for Low Back Pain: A Randomized Clinical Trial
Source: JAMA Netw Open. 2025 Jan 10;8(1):e2453807. doi: 10.1001/jamanetworkopen.2024.53807 (PMC11724347; doi:10.1001/jamanetworkopen.2024.53807)
Supplement: Supplement 3. — Nonauthor Collaborators. Healthy Lifestyle Program for Chronic Low Back Pain (HeLP) Trial working group members [file jamanetwopen-e2453807-s003.pdf]

Supplemental Online Content: Nonauthor Collaborators
*First name, last name, and suffix (if applicable) are required and will appear in PubMed.

*Group Name(s): Healthy Lifestyle Program (HeLP) for Chronic Low Back Pain Trial working group							
***First Name and Middle Initial(s)**	***Last Name**	***Suffix (eg, Jr, III)**	Academic Degrees	Institution	Location (city, state/province, country)	Role or Contribution, eg, chair, principal investigator	Group (if more than 1 Group listed in the byline) and/or Subgroup (eg, Steering Committee)
Hopin	Lee		PhD	University of Oxford	Oxford, UK	Steering Committee	
Damien	Smith		BAppSc	John Hunter Hospital Physiotherapy Department	Newcastle, NSW, Australia	Steering Committee	
John	Wiggers		PhD	Hunter New England Population Health	Newcastle, NSW, Australia	Advisor	
Karen	Gillham		MScoSc	Hunter New England Population Health	Newcastle, NSW, Australia	Advisor	
Alix	Hall		PhD	Hunter New England Population Health	Newcastle, NSW, Australia	Advisor	
Christopher	Oldmeadow		PhD	Hunter Medical Research Institute	Newcastle, NSW, Australia	Advisor	
Erin	Nolan		PhD	Hunter Medical Research Institute	Newcastle, NSW, Australia	Analyst	
Emma-Leigh	Simpson		BAppSc	John Hunter Hospital Physiotherapy Department	Newcastle, NSW, Australia	Steering Committee	intervention delivery
Martin	O'Neill		BAppSc	John Hunter and Belmont Hospital Physiotherapy Department	Newcastle, NSW, Australia	Steering Committee	intervention delivery
Catherine	Groves		BAppSc	John Hunter and Belmont Hospital Physiotherapy Department	Newcastle, NSW, Australia	Steering Committee	intervention delivery
