# Supplementary material for: Healthy Lifestyle Care vs Guideline-Based Care for Low Back Pain: A Randomized Clinical Trial
Source: JAMA Netw Open. 2025 Jan 10;8(1):e2453807. doi: 10.1001/jamanetworkopen.2024.53807 (PMC11724347; doi:10.1001/jamanetworkopen.2024.53807)
Supplement: Supplement 4. — Data Sharing Statement [file jamanetwopen-e2453807-s004.pdf]

Data Sharing Statement

Mudd. Healthy Lifestyle Care vs Guideline-Based Care for Low Back Pain. *JAMA Netw Open*. Published January 10, 2025. doi:10.1001/jamanetworkopen.2024.53807

Data
Additional Information: ACTRN12617001288314
Data available: Yes
Data types: Deidentified participant data, Data dictionary
How to access data: Data can be made available on approved requests to the corresponding author - c.williams@sydney.edu.au
When available: With publication

Supporting Documents
Document types: None

Additional Information
Who can access the data: Researchers whose proposed use of the data has been approved.
Types of analyses: Secondary use of data will be considered by the investigator team.
Mechanisms of data availability: With a signed data access agreement.
Any additional restrictions: Additional ethical approval for secondary data use may be required.
